# The Role of Mms22p in DNA Damage Response in *Candida albicans*

**DOI:** 10.1534/g3.115.021840

**Published:** 2015-10-04

**Authors:** Lan Yan, Juan Xiong, Hui Lu, Quan-zhen Lv, Qian-yao Ma, Pierre Côte, Malcolm Whiteway, Yuan-ying Jiang

**Affiliations:** * Center for New Drug Research, School of Pharmacy, Second Military Medical University, Shanghai 200433, P. R. China; † Department of Biology, Concordia University, Montreal, Quebec, H4B 1R6, Canada; ‡ Key Laboratory of the Plateau of the Environmental Damage Control, Lanzhou General Hospital of Lanzhou Military Command, Lanzhou, 730050, China; § School of Life Science and Biopharmaceutics, Shenyang Pharmaceutical University, Shenyang, Liaoning Province 110016, P.R. China

**Keywords:** genomic stability, DNA repair, replication fork, homologous recombination, *Candida albicans*

## Abstract

To ensure correct DNA replication, eukaryotes have signaling pathways that respond to replication-associated DNA damage and trigger repair. In both *Saccharomyces cerevisiae* and *Schizosaccharomyces pombe*, a complex of proteins, including the cullin protein Rtt101p and two adapter proteins Mms22p and Mms1p, is important for proper response to replication stress. We have investigated this system in *Candida albicans*. In this pathogen, Mms22p is important for recovery from DNA replication damage induced by agents including methylmethane sulfonate, camptothecin, and ionizing radiation. Although no clear ortholog of Mms1p has been identified in *C. albicans*, loss of either Mms22p or Rtt101p generates similar damage sensitivity, consistent with a common function. In *S. cerevisiae*, the Mrc1p−Csm3p−Tof1p complex stabilizes stalled replication forks and activates a replication checkpoint and interacts with Mms22p. A similar complex in *S. pombe*, consisting of the Tof1p and Csm3p orthologs Swi1p and Swi3p, along with the fission yeast Mrc1p, genetically also interacts with Mms22p. Intriguingly in *C. albicans* only Mrc1p and Csm3p appear involved in damage repair, and Mms22p is required for responding to DNA damage agents in *MRC1* or *CSM3* conditional mutants. In *C. albicans*, although the loss of *RAD57* greatly impairs response in the pathogen to many DNA-damaging agents, lethality due to camptothecin damage requires concomitant loss of Rad57p and Mms22p, suggesting that Mms22p is only essential for homologous recombination induced by camptothecin. These results establish that although *C. albicans* uses conserved cellular modules to respond to DNA damage and replication blocks, the specific details of these modules differ significantly from the *S. cerevisiae* model.

Accurate transmission of the genome from one generation to the next requires the faithful replication of the DNA. In eukaryotic organisms, the process of DNA replication is challenged by replication stresses, such as dNTP depletion caused by hydroxyurea (HU), alkylated DNA template bases induced by methylmethane sulfonate (MMS), replication fork blockage caused by the topoisomerase I inhibitor camptothecin (CPT) (Vaisica *et al.* 2011), and single-strand or double-strand breaks due to ionizing radiation (IR) (Ward 1990). To ensure the fidelity and coordinate the progression of DNA replication, this challenging process is regulated by a DNA damage response network that includes S-phase checkpoints that sense stalled replication forks and DNA damage and facilitate DNA repair processes (Harper and Elledge 2007). Mechanisms of DNA repair primarily involve homologous recombination (HR), nonhomologous end-joining, and nucleotide excision repair (Wu and Hickson 2006).

In both the budding yeast *Saccharomyces cerevisiae* and the fission yeast *Schizosaccharomyces pombe*, there is an ubiquitin-conjugating complex consisting of orthologs of the budding yeast Rtt101p, Mms22p, and Mms1p that function in the damage repair process. Loss of *Sc*Mms22p or *Sp*Mms22p increases cellular sensitivity to a range of DNA-damaging drugs that generate lesions specifically in S-phase or that directly impede DNA replication (Chang *et al.* 2002; Araki *et al.* 2003; Baldwin *et al.* 2005; Dovey and Russell 2007; Duro *et al.* 2008; Vaisica *et al.* 2011). In *S. cerevisiae*, genetic epistasis between Mms22p and Mms1p suggests that Mms22p interacts with Rtt101p via Mms1p to form a protein complex (Rtt101p−Mms1p−Mms22p) required to promote recombinational repair at stalled replication forks and that this complex is required for replication of damaged DNA (Ho *et al.* 2002; Dovey and Russell 2007; Tourriere and Pasero 2007; Duro *et al.* 2008; Zaidi *et al.* 2008; Vaisica *et al.* 2011). *Sc*Mms22p is involved in sensing replication intermediates or in the prevention of DNA damage caused by blocked replication forks.

In *S. cerevisiae*, Mms22p is important for the stable association of the fork-pausing complex (Mrc1p−Tof1p−Csm3p) when replication stress is present (Vaisica *et al.* 2011). *Sc*Mrc1p acts as a primary mediator for transducing replication fork-pausing checkpoint signals and forms a stable complex with Csm3p and Tof1p to promote sister chromatid cohesion after DNA damage (Nedelcheva *et al.* 2005). *S. pombe* Mms22p also has been confirmed to interact genetically with components of the replication fork, such as the Swi1p−Swi3p−Mrc1p complex, to restart DNA replication at stalled forks. *Sp*Mms22p functions in the stabilization of paused replication forks as well (Dovey and Russell 2007).

HR is a high-fidelity DNA repair pathway. Besides playing a critical role in accurate chromosome segregation during meiosis, HR functions in DNA repair and in the recovery of stalled or broken replication forks to ensure genomic stability. In *S. cerevisiae*, HR proceeds through either Rad51p-dependent or Rad51p-independent pathways. The Rad51p-dependent pathway of recombination, also requiring Rad52p, Rad55p, Rad57p and Rad54p, is the most efficient pathway for gene conversion and is also required for repair of most double-strand DNA breaks in mitotic cells (Johnson and Symington 1995). The Rad51p-independent pathway depends on Rad59p (Sakofsky *et al.* 2012). Budding yeast Mms22p is required for HR-mediated repair of stalled or broken DNA replication forks (Duro *et al.* 2008), whereas *S. pombe* Mms22p might block the action of HR (Dovey and Russell 2007).

Although the central role of Mms22p in the maintenance of genome integrity is well characterized in *S. cerevisiae* and in *S. pombe* (Dovey and Russell 2007; Duro *et al.* 2008; Vaisica *et al.* 2011), the orthologous protein in *Candida albicans* has not been investigated extensively. Here we reported the identification and initial characterization of the *MMS22*/*CR_00390W_A* gene in *C. albicans* as the putative ortholog of the *MMS22* gene in *S. cerevisiae*, and we identified *C1_06040W_A*, a putative ortholog of *ScRTT101* in *C. albicans*, as the Mms22p-interacting protein in the ubiquitin-conjugating complex. To test the function of Mms22p and its potential partner proteins in the fork-pausing complex in *C. albicans*, we identified *C1_11440C_A* (a putative ortholog of *ScMRC1*) (Shi *et al.* 2007), *C2_06130W_A* (a putative ortholog of *ScCSM3*), and *C5_01460W_A* (a putative ortholog of *ScTOF1*) in *C. albicans*. To further explore the involvement of Mms22p in HR repair, we characterized *C2_08110W_A* (a putative ortholog of *ScRAD57* in *C. albicans*), as previous studies have revealed that the conserved Rad51p, Rad52p, Rad54p, and Rad59p play important role in the HR in *C. albicans* (Ciudad *et al.* 2004; Garcia-Prieto *et al.* 2010; Hoot *et al.* 2011). We constructed a set of single-gene and double-gene mutants, including the conditional single gene mutants of P*_MET3_-MMS22*, P*_MET3_-MRC1*, P*_MET3_-TOF1*, and P*_MET3_-CSM3*, as well as the double-gene mutants of the aforementioned genes repressed together with *MMS22*. We also constructed the null mutants of Δ*rtt101*and Δ*rad57*, as well as the double-gene mutants of the genes deleted together with *MMS22*. Our present study shows that *Ca*Mms22p plays a vital role in preserving genome integrity during DNA replication and is important for viability after DNA replication-associated damage.

## Materials and Methods

### Media and culture conditions

Unless otherwise indicated, all the strains were grown routinely in YPD medium (*i.e.*, 1% yeast extract, 2% peptone, and 2% dextrose) at 30° with shaking overnight, diluted to an OD_600_ of 0.1−0.2, grown to logarithmic phase, and used for subsequent experiments. As indicated, synthetic complete (SC) medium (0.67% yeast nitrogen base and 2% dextrose) was supplemented with histidine (20 µg/mL), leucine (60 µg/mL), or arginine (40 µg/mL) as appropriate. For repressing conditions for the *C. albicans MET3* promoter, mutants were cultured in SC medium with 2.5 mM methionine (Met) and 2.5 mM cysteine (Cys) (SC-Met^+^/Cys^+^). To induce the *MET3* promoter, mutants were grown in SC medium without Met and Cys (SC-Met^−^/Cys^−^) (Care *et al.* 1999).

### Strain constructions

The *C. albicans* strains used in this study are listed in Table 1. The oligonucleotides used in this study are listed in supporting information, Table S1. A detailed version of all the strain constructions is provided as supplementary information (File S1). In summary, to generate the conditional *C. albicans MMS22* mutant, one allele was placed under the control of the Met/Cys-repressible *MET3* promoter (Care *et al.* 1999), and the other allele was disrupted with the *Candida dubliniensis HIS1* marker (Noble and Johnson 2005). To achieve this, the *SAT1-MET3p* cassette from plasmid pFA-SAT1-MET3p (Schaub *et al.* 2006) was amplified by using the primers oLY152 and oLY153 to generate a *SAT1-MET3p-MMS22* cassette with 100 bp of homology to the 5′ upstream region of *MMS22* and 100 bp of homology to the beginning of the *MMS22* open reading frame. The sequence of *C. dubliniensis HIS1* from plasmid pSN52 (Noble and Johnson 2005) was fused to flanking homology to the 5′ upstream and 3′ downstream regions of the *MMS22* gene to generate a *mms22*∆::*C.d.HIS1* disruption cassette. *C. albicans* SN152 was then transformed with the *mms22*∆::*C.d.HIS1* cassette to generate strains CaLY8 (*MMS22*/*mms22*::*C.d.HIS1*) and CaLY226 (*MET3p-MMS22*/*mms22*::*C.d.HIS1*).

**Table 1:** Strains used in this study

Strain Name	Strain Ref	Parental Strain	Key Genotype	Reference
SN152	WT	SC5314	arg4/arg4 leu2/leu2 his1/his1 URA3/ura3::imm434;IRO1/iro1::imm434	(Noble and Johnson 2005)
	CaLY8	SN152	orf19.7494::HIS1/ORF19.7494 arg4/arg4 leu2/leu2 his1/his1 URA3/ura3::imm434 IRO1/iro1::imm434	This study
P_MET3_-*MMS22*	CaLY226	CaLY8	orf19.7494::HIS1/SAT1-MET3p-ORF19.7494 arg4/arg4 leu2/leu2 his1/his1 URA3/ura3::imm434 IRO1/iro1::imm434	This study
	CaLY219	SN152	ORF19.4136/orf19.4136::LEU2 arg4/arg4 leu2/leu2 his1/his1 URA3/ura3::imm434 IRO1/iro1::imm434	This study
P_MET3_-*TOF1*	CaLY337	CaLY219	orf19.4136::LEU2/ARG-MET3p-ORF19.4136 arg4/arg4 leu2/leu2 his1/his1 URA3/ura3::imm434 IRO1/iro1::imm434	This study
	CaLY220	SN152	ORF19.4105/orf19.4105::LEU2 arg4/arg4 leu2/leu2 his1/his1 URA3/ura3::imm434 IRO1/iro1::imm434	This study
P_MET3_-*CSM3*	CaLY249	CaLY220	orf19.4105::LEU2/ARG4-MET3p-ORF19.4105 arg4/arg4; leu2/leu2; his1/his1; URA3/ura3::imm434;IRO1/iro1::imm434	This study
	CaLY222	SN152	ORF19.658/orf19.658::LEU2 arg4/arg4 leu2/leu2 his1/his1 URA3/ura3::imm434 IRO1/iro1::imm434	This study
P_MET3_-*MRC1*	CaLY316	CaLY222	orf19.658::LEU2/ARG4-MET3p-CaORF19.658 arg4/arg4 leu2/leu2 his1/his1 URA3/ura3::imm434 IRO1/iro1::imm434	This study
	CaLY223	SN152	ORF19.2174/orf19.2174::LEU2 arg4/arg4 leu2/leu2 his1/his1 URA3/ura3::imm434 IRO1/iro1::imm434	This study
Δ*rad57*	CaLY235	CaLY223	orf19.2174::LEU2/orf19.2174::ARG4 arg4/arg4 leu2/leu2 his1/his1 URA3/ura3::imm434 IRO1/iro1::imm434	This study
	CaLY224	SN152	ORF19.2440/orf19.2440::LEU2 arg4/arg4 leu2/leu2 his1/his1 URA3/ura3::imm434 IRO1/iro1::imm434	This study
Δ*rtt101*	CaLY236	CaLY224	orf19.2440::LEU2/orf19.2440::ARG4 arg4/arg4 leu2/leu2 his1/his1 URA3/ura3::imm434 IRO1/iro1::imm434	This study
	CaLY228	CaLY226	ORF19.658/orf19.658::LEU2 orf19.7494::HIS1/SAT1-MET3p-ORF19.7494 arg4/arg4 leu2/leu2 his1/his1 URA3/ura3::imm434 IRO1/iro1::imm434	This study
P_MET3_-*MMS22*/P_MET3_-*MRC1*	CaLY251	CaLY228	orf19.658::LEU2/ARG4-MET3p-CaORF19.658 orf19.7494::HIS1/SAT1-MET3p-ORF19.7494 arg4/arg4 leu2/leu2 his1/his1 URA3/ura3::imm434 IRO1/iro1::imm434	This study
	CaLY234	CaLY226	ORF19.4105/orf19.4105::LEU2 orf19.7494::HIS1/SAT1-MET3p-ORF19.7494 arg4/arg4 leu2/leu2 his1/his1 URA3/ura3::imm434 IRO1/iro1::imm434	This study
P_MET3_-*MMS22*/P_MET3_-*CSM3*	CaLY246	CaLY234	orf19.4105::LEU2/ARG4-MET3p-ORF19.4105 orf19.7494::HIS1/SAT1-MET3p-ORF19.7494 arg4/arg4 leu2/leu2 his1/his1 URA3/ura3::imm434 IRO1/iro1::imm434	This study
	CaLY238	CaLY226	ORF19.2174/orf19.2174::LEU2 orf19.7494::HIS1/SAT1-MET3p-ORF19.7494 arg4/arg4 leu2/leu2 his1/his1 URA3/ura3::imm434 IRO1/iro1::imm434	This study
P_MET3_-*MMS22*/Δ*rad57*	CaLY242	CaLY238	orf19.2174::LEU2/orf19.2174::ARG4 orf19.7494::HIS1/SAT1-MET3p-ORF19.7494 arg4/arg4 leu2/leu2 his1/his1 URA3/ura3::imm434 IRO1/iro1::imm434	This study
	CaLY240	CaLY226	ORF19.2440/orf19.2440::LEU2 orf19.7494::HIS1/SAT1-MET3p-ORF19.7494 arg4/arg4 leu2/leu2 his1/his1 URA3/ura3::imm434 IRO1/iro1::imm434	This study
P_MET3_-*MMS22*/Δ*rtt101*	CaLY244	CaLY240	orf19.2440::LEU2/orf19.2440::ARG4 orf19.7494::HIS1/SAT1-MET3p-ORF19.7494 arg4/arg4 leu2/leu2 his1/his1 URA3/ura3::imm434 IRO1/iro1::imm434	This study

WT, wild type.

A similar strategy was used to disrupt the *TOF1* gene. To summarize, a conditional *TOF1* mutant was generated in SN152 by insertion of the *MET3* promoter before the ATG start codon of the *TOF1* gene. The *ARG4-MET3p* cassette from plasmid pFA-ARG4-MET3p (Schaub *et al.* 2006) was amplified by using the primers oLY312 and oLY313 and then fused with the upstream region and the beginning of the *TOF1* open reading frame to generate an *ARG4-MET3p-TOF1* cassette. The *Candida maltosa LEU2* sequence from plasmid pSN40 (Noble and Johnson 2005) was fused to flanking homology to the 5′ upstream and 3′ downstream regions of the *TOF1* gene to generate a *tof1*∆::*C.m.LEU2* disruption cassette. *C. albicans* SN152 was then transformed with the *tof1*∆::*C.m.LEU2* cassette to generate strains CaLY219 (*TOF1*/*tof1*::*C.m.LEU2*) and CaLY337 (*MET3p-TOF1*/*tof1*::*C.m.LEU2*). On the basis of the same logic, the conditional disruptions of *CSM3* (CaLY249, *MET3p-CSM3*/*csm3*::*C.m.LEU2*) and *MRC1* (CaLY316, *MET3p-MRC1*/*mrc1*::*C.m.LEU2*) were constructed.

The entire encoding sequences of *RAD57* and *RTT101* were deleted from the wild-type strain SN152 by two-step HR by the use of a fusion−polymerase chain reaction (PCR)-based strategy (Noble and Johnson 2005). To summarize, the two *RAD57* alleles were disrupted sequentially with *rad57*∆::*C.m.LEU2* and *rad57*∆::*C.d.ARG4* disruption cassettes to create the homozygous *rad57* null mutant (CaLY235). The two *RTT101* alleles were replaced sequentially with *rtt101*∆::*C.m.LEU2* and *rtt101*∆::*C.d.ARG4* disruption cassettes to create the homozygous *rtt101* null mutant (CaLY236).

The double-gene mutants of P*_MET3_-MMS22*/Δ*csm3*, P*_MET3_-MMS22*/Δ*rad57*, and P*_MET3_-MMS22*/Δ*rtt101* were generated by transforming the conditional *MMS22* mutant (strain CaLY226) with *csm3*∆::*C.m.LEU2* (creating CaLY234), *rtt101*∆::*C.m.LEU2* (creating CaLY240), or *rad57*∆::*C.m.LEU2* (creating CaLY238), followed by *csm3*∆::*C.d.ARG4* (creating CaLY246), *rtt101*∆::*C.d.ARG4* (creating CaLY244), or *rad57*∆::*C.d.ARG4* (creating CaLY242), respectively. The double mutant of P*_MET3_-MMS22*/P*_MET3_-MRC1* was generated by transforming strain CaLY226 with *mrc1*∆::*C.m.LEU2* (creating CaLY228), followed by *ARG4-MET3p-MRC1* cassette (creating CaLY251). All transformants were checked for correct genome integration by genomic PCR.

### Flow cytometry

*C. albicans* cells derived from an exponentially growing of culture in SC-Met^+^/Cys^+^ medium were arrested with 0.01% MMS or 20 mM HU for 4 hr at 30° with sampling every 2 hr. Cells were then washed to remove MMS or HU, resuspended in SC-Met^+^/Cys^+^ medium, and incubated for an additional 4 hr with sampling every 2 hr. Samples of 3 mL were fixed with 70% ethanol overnight at 4°, washed with phosphate-buffered saline (pH = 7.4) and digested with 1 mg/mL RNase A for 1 hr at 37° to remove RNA. Cells were then stained with 50 mg/mL of propidium iodide for at least 4 hr at room temperature. The DNA content of 5 × 10^4^ cells was monitored by fluorescence-activated cell sorting (FACS) analysis using a flow cytometer (FACSCALIBUR; BD Bioscience) and analyzed by Cellquest software (BD Bioscience). The vertical axis is cell counts and the horizontal axis is nuclear fluorescence. Three independent experiments were performed.

### DNA damage sensitivity assays

Mid-log phase cultures were adjusted to 5 × 10^6^ cells/mL, fivefold serially diluted, and spotted onto solid SC-Met^+^/Cys^+^ or SC-Met^−^/Cys^−^ medium, which contains the indicated concentration of MMS, CPT, or HU. Alternatively, serial dilutions of cells were spotted onto solid SC-Met^+^/Cys^+^ or SC-Met^−^/Cys^−^ medium for irradiating with the indicated dose of IR. Growth of cells was detected after a 48-hr incubation period at 30°.

For testing the survival of cells with exposure to MMS or HU, mid-log phase *C. albicans* cells were cultured in SC-Met^+^/Cys^+^ medium containing 0.005% MMS or 20 mM HU for 12 hr. At the indicated time points, samples were pooled, washed, and a range of 500−1000 cells were spread onto solid SC-Met^+^/Cys^+^ medium in triplicate. The number of colonies was counted following incubation at 30° for 2 d. The percentage of survival with untreated normalized to 100% at the indicated time points were calculated. Three independent experiments were performed.

### Morphogenesis analysis

Mid-log phase *C. albicans* cells were adjusted to 1 × 10^3^ cells/mL. A total of 100 µL of each strain culture were spread onto solid SC-Met^+^/Cys^+^ or SC-Met^−^/Cys^−^ medium, incubated at 30° for 3−4 d, and photographed. Meanwhile, mid-log cultures adjusted to 1 × 10^3^ cells/mL were grown for another 9 hr in liquid SC-Met^+^/Cys^+^ or SC-Met^−^/Cys^−^ medium with shaking at 30°, then photographed with a EVOS X1 microscope (Life Technologies).

### Alignments

We aligned Mms22p, Tof1p, Csm3p, Mrc1p, plus Rad57p primary amino sequences in *S. cerevisiae*, *C. albicans*, and *S. pombe*. Rtt101p sequences were aligned in *S. cerevisiae* and *C. albicans*. Multiple protein sequence alignments were performed with the MAFFT web application (http://mafft.cbrc.jp/alignment/server/) and visualized with Jalviewer (Version 2.8). The primary amino sequences of the *S. cerevisiae*, *C. albicans*, and *S. pombe* proteins were downloaded from the Fungal Orthogroups Repository (http://www.broadinstitute.org/cgi-bin/regev/orthogroups) hosted by the Broad Institute, MIT.

### Data availability

Strains are available upon request. File S1 contains detailed descriptions of all supplemental files.

## Results

### Identification of Mms22p and its partner proteins in *C. albicans*

We used the Fungal Orthogroups Repository (Wapinski *et al.* 2007) to identify that the *C. albicans CR_00390W_A* gene is orthologous to both the *S. cerevisiae MMS22* gene and the *S. pombe mms22* gene. *C. albicans CR_00390W_A* encodes a protein with 1704 amino acids (molecular weight 196.9 kDa). When this protein is aligned with *Sc*Mms22p, it showed 5% identity and 19% similarity (Table 2; Figure S1A). We predicted that *CaCR_00390W_A* is a functional ortholog of *ScMMS22* and classified *CaCR_00390W_A* as *CaMMS22*.

**Table 2:** *C. albicans* proteins studied in this study and their orthologs in *S. cerevisiae* and in *S. pombe*

Species	Proteins and Orthologs						
*C. albicans*	Mms22p/*CR_00390W_A*	Rtt101p/*C1_06040W_A*	n/a	Mrc1p/*C1_11440C_A*	Csm3p*/C2_06130W_A*	Tof1p*/C5_01460W_A*	Rad57p*/C2_08110W_A*
*S. cerevisiae*	Mms22p/Ylr320wp	Rtt101p/Yjl047c	Mms1p/Ypr164w	Mrc1p/Ycl061c	Csm3p/Ymr048w	Tof1p/Ynl273w	Rad57p/Ydr004w
*S. pombe*	Mms22p/ SPAC6B12.02c	n/a	Mms1p/SPAC3H8.05c	Mrc1p/SPAC694.06c	Swi3p/SPBC30D10.04	Swi1p/SPBC216.06c	Rhp57p/SPAC20H4.07
	Percentage of Sequence Homology and Identity of *C. albicans* Proteins Aligned with *S. cerevisiae* Counterparts						
Identity	5%	5%	n/a	7%	9%	6%	5%
Similarity	19%	18%	n/a	23%	22%	20%	16%

n/a, not available.

Similarly, we identified *C. albicans C5_01460W_A* and *C2_06130W_A*, as orthologous to *ScTOF1* and *ScCSM3*, as well as *Spswi1* and *Spswi3*, respectively, by using the Fungal Orthogroups Repository. Alignment of *ScTOF1* and *C. albicans C5_01460W_A* indicated 6% identity and 20% similarity over their full-length sequences (Table 2; Figure S1B), and 9 and 22% between *ScCSM3* and *C. albicans C2_06130W_A* (Table 2; Figure S1C), respectively. We named *C. albicans C5_01460W_A* and *C2_06130W_A* as *CaTOF1* and *CaCSM3*. We also identified *C2_08110W_A*/*CaRAD57* as the ortholog to *ScRAD57* with 5% identity and 16% similarity, and to *Sprph57* with 5% identity and 15% similarity (Table 2; Figure S1E). However, we failed to find a gene homologous to *ScRAD55* or *Sprph55* in *C. albicans*. To address the function of possible Rtt101p and Mms1p paralogs in *C. albicans*, the Fungal Orthogroups Repository was used to identify *C1_06040W_A* as a ortholog to *ScRTT101* with 5% identity and 18% similarity (Table 2; Figure S1F), whereas no homologous gene to *ScMMS1* was found. *C1_11440C_A* has previously been reported as orthologous to *ScMRC1* (Shi *et al.* 2007).

### Generation of conditional mutants of *mms22* and its partner genes in *C. albicans*

To test the function of *MMS22* in *C. albicans*, the first allele was replaced with the *mms22*∆::*C.d.HIS1* cassette (strain CaLY8) using the fusion−PCR-based strategy in strain SN152 (Noble and Johnson 2005). A conditional *MMS22* mutant was constructed from CaLY8 in which the single remaining copy of *MMS22* was placed under the control of the *MET3* promoter (strain CaLY226; Figure S2). *MMS22* mRNA levels in the wild-type SN152 and the P*_MET3_-MMS22* strain grown under nonrepressed and repressed conditions were analyzed by relative quantitative real-time PCR. The transcription level of *CaACT1* was used as a standard for normalization. *MMS22* mRNA levels in the repressed P*_MET3_-MMS22* strain were 10-fold lower than in the wild-type strain after 12 hr of growth in SC-Met^+^/Cys^+^ medium, whereas the transcription level of *MMS22* is similar to the wild type strain in SC-Met^−^/Cys^−^ medium. The conditional expression of the *MMS22* mutant allowed us to study the function of this gene in *C. albicans*.

Since the budding yeast Mms22p and the fission yeast *Sp*Mms22p genetically interact with the fork-pausing complex to stabilize the replisome during replication stress, we constructed conditional single-gene mutants of regulated expression of *TOF1*, *MRC1*, or *CSM3*, and the conditional double-gene mutants of P*_MET3_-MMS22*/P*_MET3_-MRC1* and P*_MET3_-MMS22*/P*_MET3_-CSM3* (Figure S3) to explore a potential link between Mms22p and Tof1p, Mrc1p, or Csm3p in *C. albicans*. The regulated expression of the target genes was confirmed by quantitative real-time PCR (data not shown).

To probe the function of the *RTT101* gene and the relationship between the *RTT101* gene and the *MMS22* gene in *C. albicans*, both alleles of *RTT101* were deleted in the wild-type SN152 and P*_MET3_-MMS22* strains, to generate ∆*rtt101* and P*_MET3_-MMS22*/∆*rtt101* mutants (Figure S4). Similarly, to explore whether *CaMMS22* is involved in HR repair, both alleles of *CaRAD57* in either the wild-type SN152 or the P*_MET3_-MMS22* mutant were deleted, respectively, to obtain ∆*rad57* and P*_MET3_-MMS22*/∆*rad57* mutants (Figure S4).

### Mms22p is important for the recovery from a disturbed DNA replication in *C. albicans*

**Figure 1:**
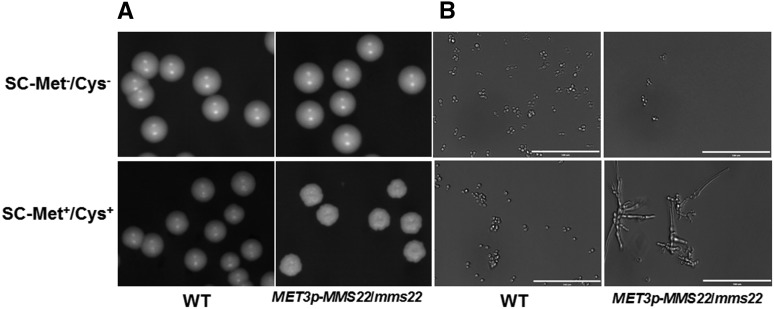
Colony and single-cell morphology of the parental strain SN152 (wild type; WT) and the P*_MET3_-MMS22* mutant strains. (A) Colony morphology after 3 d of growth on solid SC-Met^−^/Cys^−^ and SC-Met^+^/Cys^+^ media at 30° are shown. (B) Cells from an overnight liquid SC-Met^−^/Cys^−^ and SC-Met^+^/Cys^+^ culture at 30° were examined under microscope. Bar = 100 μm.

In contrast to the wild-type strain, in which yeast cells formed smooth, domed colonies and separated readily after cytokinesis, we found that the P*_MET3_-MMS22* strain grew normally under nonrepressing conditions but formed rough, flattened colonies (Figure 1A) and elongated cells (Figure 1B) when the *MET3* promoter was repressed in SC-Met^+^/Cys^+^ medium, even in the absence of any genotoxic stress. In general, in response to cell-cycle arrest in *C. albicans*, a filamentous cell type with characteristics of both pseudohyphae and true hyphae appears (Berman 2006). The elongated cells suggested that the repressed *MMS22* mutant could be defective in DNA replication or were unable to repair DNA breaks appearing spontaneously during replication.

**Figure 2:**
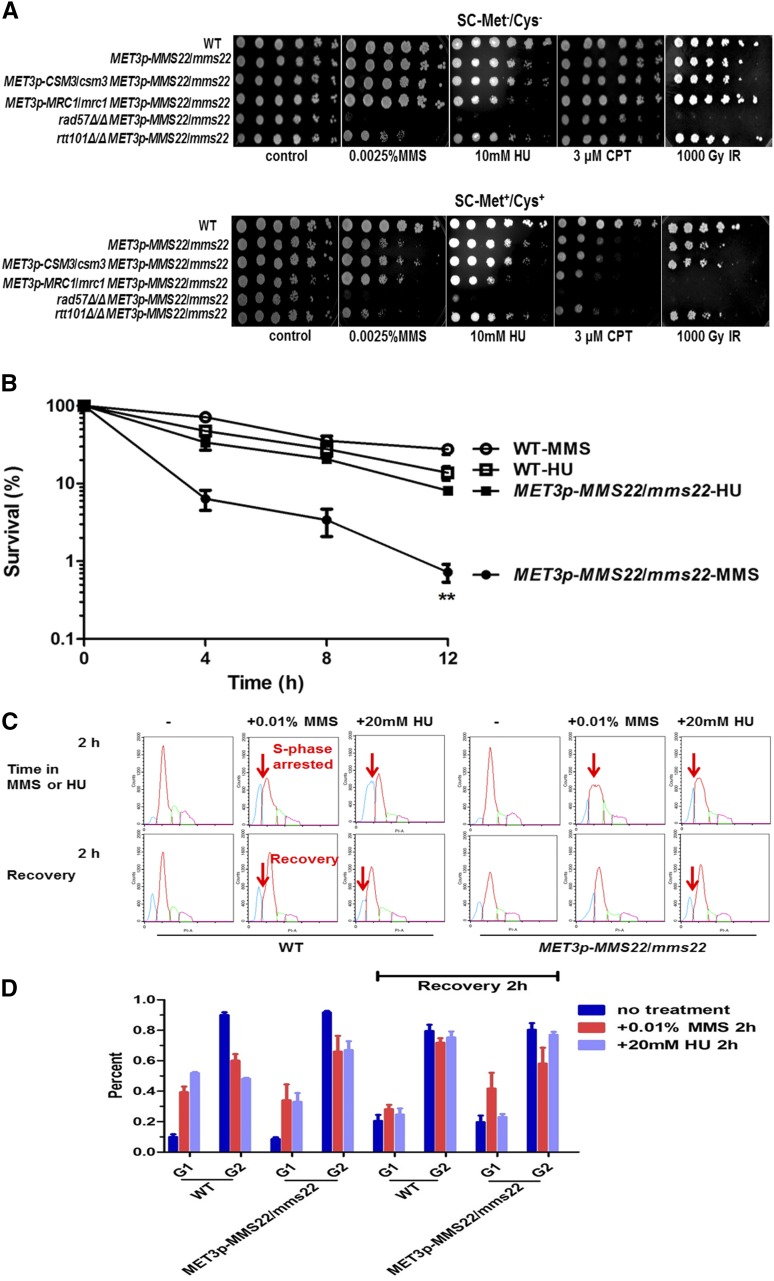
Sensitivity of the mutant strains to DNA-damaging agents. (A) Spot assays comparing the sensitivity of the wild-type SN152 (WT) and the mutant strains. Cells were grown as described in the section *Materials and Methods*, diluted, and spotted onto plates with the indicated concentrations of MMS, HU, CPT, and indicated dose of IR. The strains were cultured on solid SC-Met^−^/Cys^−^ and SC-Met^+^/Cys^+^ media independently, and photographed after 2 d growth at 30°. (B) Survival curves of *MET3p-MMS22/mms22* mutant exposed to 0.01% MMS or 20 mM HU for 12 hr. A total of 500–1000 cells were plated on SC-Met^+^/Cys^+^ agar in triplicate. Colony numbers were counted following incubation at 30° for 2 d. The percentage of survival with untreated normalized to 100% at the indicated time points are shown. The mean and the standard deviation of three independent experiments are plotted. (C) DNA content analyzed by flow cytometry. Cells derived from an exponentially culture in SC-Met^+^/Cys^+^ medium were arrested with 0.01% MMS or 20 mM HU for 4 hr, and then washed and released into SC-Met^+^/Cys^+^ medium without MMS or HU for another 4 hr. Samples were collected every 2 hr. One represented cell-cycle progress in the mutants is shown. The vertical axis is cell counts and the horizontal axis is nuclear fluorescence. Blue and red curves indicate cells in G1 and G2 phases, respectively. Green and purple curves indicate DNA contents of aggregated cells which have not been analyzed. Arrows indicate the S-phase arrested and the recovery points. (D) Percentage of G1/G1+G2 and G2/G1+G2 of three independent experiments are shown.

*S. cerevisiae mms22*Δ and *S. pombe mms22*Δ mutants are sensitive to MMS, HU, and CPT but less sensitive (or resistant) to IR (Table 3) (Chang *et al.* 2002; Araki *et al.* 2003; Dovey and Russell 2007). We assessed the sensitivity of the P*_MET3_-MMS22* mutant strain to various DNA damaging agents. The P*_MET3_-MMS22* strain showed increased sensitivity to MMS and CPT, and intriguingly showed enhanced sensitivity to IR, but not to HU, when the gene is shut off by growth in SC-Met^+^/Cys^+^ medium (Figure 2A). To further confirm the differential sensitivity to MMS and HU in the P*_MET3_-MMS22* strain in the repressed condition, we checked the viability of the strains during a 12-hr period of MMS or HU exposure (Figure 2B). MMS began to significantly inhibit growth of the P*_MET3_-MMS22* strain relative to wild type after 4 hr of treatment, whereas HU affected viability in both the wild-type and P*_MET3_-MMS22* strains similarly. These results indicated that the *MMS22* gene is required for MMS resistance even during short-term MMS exposure in *C. albicans*, but is not needed for HU resistance, in contrast to *S. cerevisiae MMS22* and *S. pombe mms22*, which are required for both MMS and HU resistance (Bennett *et al.* 2001; Dovey and Russell 2007).

**Table 3:** Morphology and sensitivity to DNA damage agents of the mutants in the three species

Species	P*_MET3_-MMS22*	*RTT101*Δ/Δ	P*_MET3_-MMS22 RTT101*Δ/Δ	P*_MET3_-MRC1*	P*_MET3_-MMS22* P*_MET3_-MRC1*	P*_MET3_-CSM3*	P*_MET3_-MMS22* P*_MET3_-CSM3*	P*_MET3_-TOF1*		*RAD57*Δ/Δ	P*_MET3_-MMS22 RAD57*Δ/Δ
*C. albicans*											
Morphology	Elongated	Wild type	Elongated	Elongated	Elongated	Elongated	Elongated	Wild type		Wild type	Elongated
MMS sensitivity	Increased	Increased	More increased	Increased	Less increased	Increased	Less increased	No change		Increased	Increased
HU sensitivity	No change	No change	No change	Less increased	Less increased	Less increased	Less increased	No change		Increased	Increased
CPT sensitivity	Increased	Increased	Increased	Increased	More increased	Increased	More increased	No change		Moderate increased	More increased
IR sensitivity	Increased	Increased	Increased	Increased	More increased	Increased	More increased	No change		Increased	Increased
*S. cerevisiae*	*mms22*Δ										
MMS sensitivity	Increased										
HU sensitivity	Increased										
CPT sensitivity	Increased										
IR sensitivity	Moderate increased										
*S. pombe*	*mms22*Δ			*mrc1*Δ	*mms22*Δ *mrc1*Δ	*swi3*Δ	*mms22*Δ *swi3*Δ	*swi1*Δ	*mms22*Δ swi1Δ	*rhp57*Δ	*mms22*Δ*rhp57*Δ
Morphology	Elongated										
MMS sensitivity	Increased			No change	More increased	No change	Less increased	No change	Less increased	Increased	More increased
HU sensitivity	Increased			Increased	More increased	Increased	Less increased	Increased	Less increased	Increased	More increased
CPT sensitivity	Increased			No change	More increased	No change	Less increased	No change	Less increased	Increased	More increased
IR sensitivity	Resistant									Increased	More increased

We then used flow cytometry (FACS) to examine the changes in cell-cycle progress in the P*_MET3_-MMS22* strain during treatment with MMS or HU for 4 hr, and during a following 4-hr recovery period. The wild-type and P*_MET3_-MMS22* strains showed similar behavior during the whole cell cycle with the treatment of MMS or HU, and arrested in S phase (Figure 2C, S-phase arrested arrows). After removal of either agent followed by culturing in fresh media, the wild-type strain progressed through the cell cycle within 2 hr, as evidenced by the re-emergence of cells in the G2 phase (Figure 2C, recovery arrows) and the increase in the percentage of G2 cells (Figure 2D). By contrast, the P*_MET3_-MMS22* strain treated with MMS remained in S phase with only one peak and did not proceed into the cell cycle within 2 hr (Figure 2C, no recovery arrow) and even 4 hr (data not shown) without the increased percentage of G2 cells (Figure 2D). However, the P*_MET3_-MMS22* strain treated with HU re-entered the cell cycle 2 hr upon removal of the HU, as evidenced by the re-emergence of G2-phase peak and the increase in the percentage of G2 cells, similar to the wild-type strain (Figure 2C, recovery arrows; Figure 2D). These results suggested that during decreased expression of *MMS22*, cells were unable to recover from arrest triggered by MMS. Thus, our data suggest that *C. albicans* Mms22p is essential for recovery from the DNA replication damage induced by MMS (and potentially CPT and IR) and that the repression of *MMS22* caused an abnormal cell cycle after recovery from replication stress.

### Mms22p is required for responding to DNA damage agents in *MRC1* or *CSM3* conditional mutants’ fork-pausing complex

To explore a potential link between Mms22p and Tof1p (*S. pombe* Swi1p), Mrc1p or Csm3p (*S. pombe* Swi3p) in *C. albicans*, the conditional single-gene mutants permitting regulated expression of *TOF1*, *MRC1*, or *CSM3*, and the conditional double-gene mutants of P*_MET3_-MMS22*/P*_MET3_-MRC1*, P*_MET3_-MMS22*/P*_MET3_-CSM3* were constructed.

**Figure 3:**
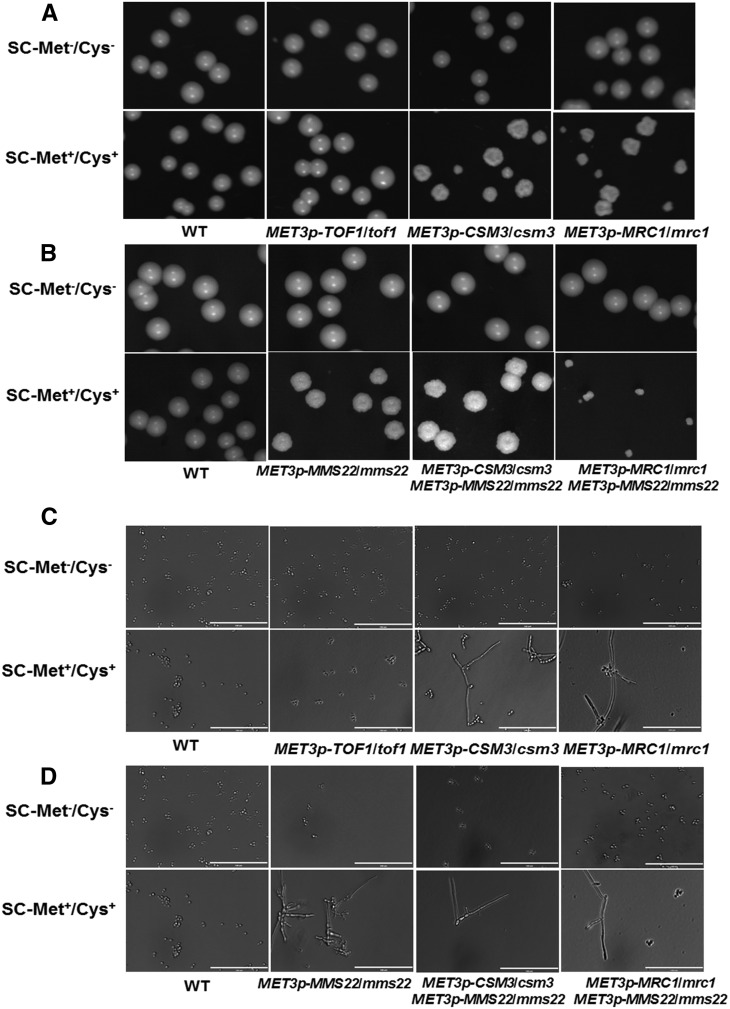
Colony and single cell morphology of the wild-type SN152 (WT) and the mutant strains. (A, B) Colony morphology after 2 d of growth on solid SC-Met^−^/Cys^−^ or SC-Met^+^/Cys^+^ medium at 30° were shown. (C, D) Cells from an overnight liquid SC-Met^−^/Cys^−^ or SC-Met^+^/Cys^+^ culture at 30° were examined under microscope. Bar = 100 μm.

**Figure 4:**
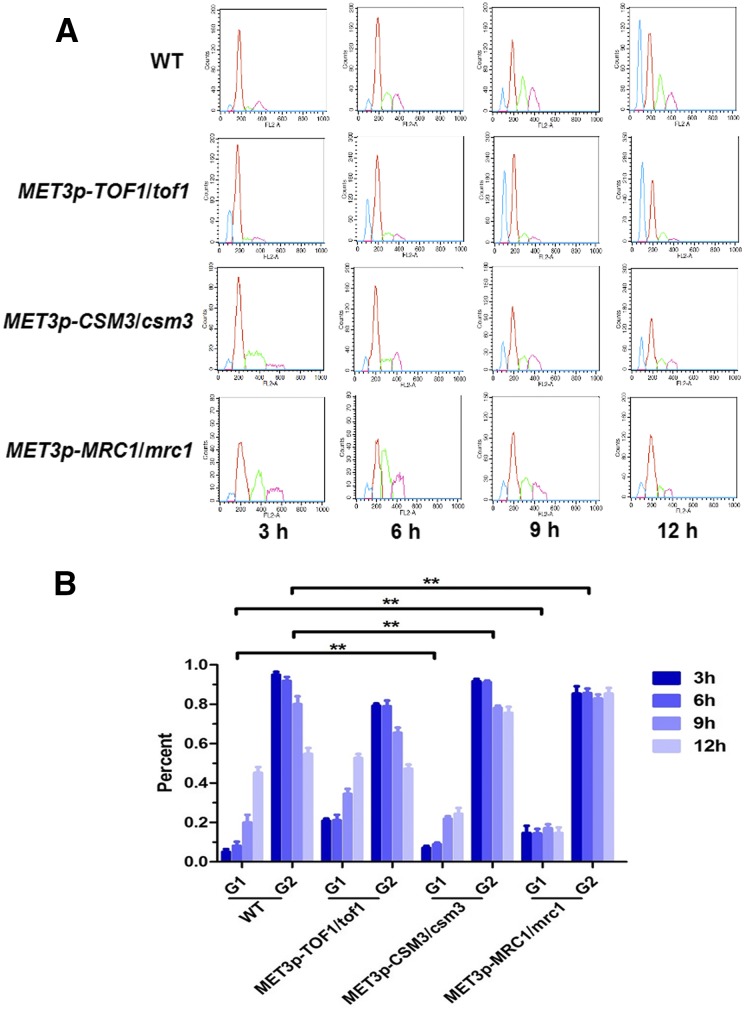
DNA content analyzed by flow cytometry. Cells derived from an exponentially growing of culture in SC-Met^+^/Cys^+^ medium for 12 hr. Samples were collected every 3 hr. (A) One represented cell-cycle progress in the mutants is shown. The vertical axis is cell counts and the horizontal axis is nuclear fluorescence. Blue and red curves indicate cells in G1 and G2 phases, respectively. Green and purple curves indicate DNA contents of aggregated cells which have not been analyzed. (B) Percentage of G1/G1+G2 and G2/G1+G2 of three independent experiments are shown. **P < 0.01 when compared with the counterpart phase of the wild type.

In the absence of any genotoxic stresses, repression of *C. albicans CSM3* or *MRC1* produced viable colonies that were rough, flatted, and small in size compared with the wild-type colonies (Figure 3A). The single cells also displayed an elongated phenotype (Figure 3C). These were consistent with the FACS results, which revealed an accumulation of P*_MET3_-MRC1* or P*_MET3_-CSM3* mutants arrested in the G2 phase that can’t complete mitosis within 12 hr in the repressive conditions (Figure 4). By contrast, the repression of *TOF1* generated similar colony and cellular morphology to the wild type (Figure 3, A and C), and cells progressed through the cell cycle and completed mitosis within 12 hr normally (Figure 4). Strikingly, combined repression of both *MMS22* and *MRC1* led to significantly smaller colonies with wrinkled edges (Figure 3B) and generated elongated cells (Figure 3D), whereas combined repression of both *MMS22* and *CSM3* generated similar morphological phenotypes to those in each independent shut off (Figure 3, B and D). Because the filamentous cells can be indicative of DNA replication defects, our results suggest that the loss of *MRC1* or *CSM3* may result in defects in either DNA replication or in the repair of DNA breaks that arise spontaneously during DNA replication. The absence of *MMS22* exacerbated the defect in the *mrc1* mutant in *C. albicans*, but interestingly not in the *csm3* mutant.

**Figure 5:**
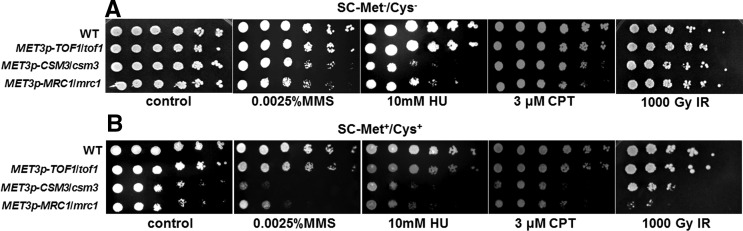
Sensitivity of the mutant strains to DNA-damaging agents. Spot assays comparing the sensitivity of the wild-type SN152 (WT) and the mutant strains. Cells were grown as described in the section *Materials and Methods*, diluted, and spotted onto plates with the indicated concentrations of MMS, HU, CPT, and indicated dose of IR. The strains were cultured on solid SC-Met^−^/Cys^−^ (A) and SC-Met^+^/Cys^+^ (B) media independently and photographed after 2 d of growth at 30°.

Repression of either *MRC1* or *CSM3* caused increased sensitivity to MMS, CPT, IR, and less sensitivity to HU, whereas the repression of *TOF1* did not affect cellular sensitivity to these agents (Figure 5; Table 3). These data suggest that these three proteins might have different function in checkpoint control or in DNA replication. By contrast, Mrc1p is essential in fork-pausing in *S. cerevisiae* but dispensable in *S. pombe* (Bennett *et al.* 2001; Calzada *et al.* 2005; Dovey and Russell 2007). Moreover, the repression of *MMS22* led to a partial rescue of the sensitivity of the P*_MET3_-CSM3* or P*_MET3_-MRC1* mutant to MMS and HU, whereas the repression of *MMS22* caused increased the sensitivity to CPT and IR of the P*_MET3_-MRC1* mutant (Figure 2A; Table 3), supporting the idea that Mms22p is required for responding to paused replication forks.

### Mms22p is required for responding to CPT in the *rad57*-null mutants

The *S. cerevisiae* Rad55p-Rad57p complex and the *S. pombe* Rhp55p-Rhp57p complex have unique nonredundant functions in recombination, and mutations in any one of these components can lead to recombination defects, chromosomal instability, sensitivity to DNA damage, and meiotic defects (Khasanov *et al.* 2008). Because *S. pombe mms22* is indispensable for replication-associated DNA damage that is repaired by HR, and the *mms22*Δ/*rph57*Δ double mutant displayed additive growth deficiencies and DNA damage sensitivites (Table 3) (Dovey and Russell 2007; Yokoyama *et al.* 2007), we investigated a similar interaction of *CaMMS22* with the HR genes in *C. albicans*.

**Figure 6:**
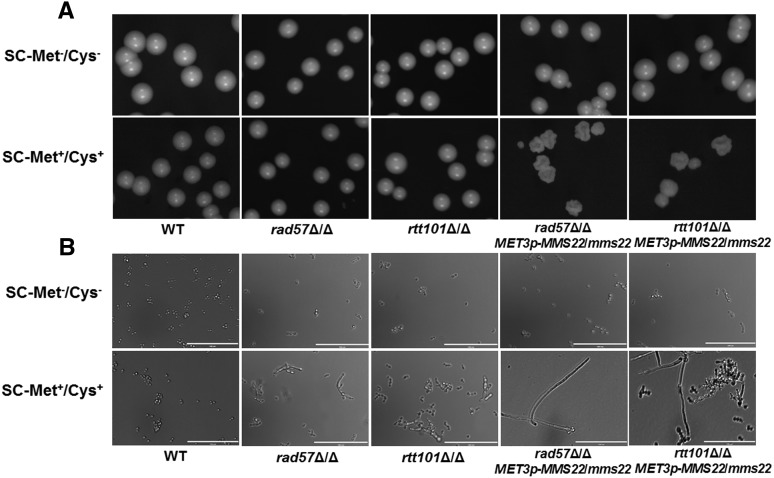
Colony and single-cell morphology of the parental strain SN152 (WT) and the indicated mutant strains. (A) Colony morphology after 2 d of growth on solid SC-Met^−^/Cys^−^ and SC-Met^+^/Cys^+^ media are shown. (B) Cells from an overnight liquid SC-Met^−^/Cys^−^ and SC-Met^+^/Cys^+^ culture at 30° were examined under a microscope. Bar = 100 μm.

**Figure 7:**
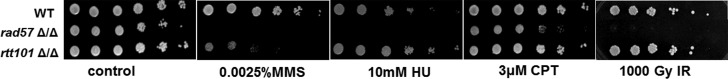
Sensitivity of the mutant strains to DNA-damaging agents. Spot assays comparing the sensitivity of the wild-type SN152 (WT) and the mutant strains. Cells were grown as described in the section *Materials and Methods*, diluted, and spotted onto plates with the indicated concentrations of MMS, HU, CPT, and indicated dose of IR. The strains were cultured on solid SC-Met^−^/Cys^−^ medium and photographed after 2 d growth at 30°.

The *rad57*∆/∆ cells formed wild-type colonies on solid media. As well, they grew as yeast cells in liquid media (Figure 6). The *rad57* null mutant was highly sensitive to the presence of DNA damaging agents such as MMS, HU, and IR (Figure 7, Figure 2A in SC-Met^−^/Cys^−^ medium). Intriguingly, the mutant showed only a slight sensitivity to CPT in comparison with the wild type, but the P*_MET3_-MMS22*/∆*rad57* strain showed high sensitivity to CPT after *MMS22* promoter shut-off (Figure 2A in SC-Met^+^/Cys^+^ medium; Table 3). These data suggested that *RAD57* is critical for responding to MMS, HU, or IR damage in *C. albicans* but is only essential for CPT damage repair in the absence of *MMS22*. This requirement of Rad57p for DNA repair in either the P*_MET3_-MMS22* mutant or the wild type strain indicated that in the absence or the presence of *MMS22*, cells could experience DNA damage that is repaired by HR.

### Mms22p and Rtt101p promote replication through damaged DNA

In budding yeast, Mms22p interacts with Rtt101p, bridged by the DNA repair protein Mms1p, and is recruited to ubiquitinate a currently unidentified substrate (or substrates) in the DNA repair process (Zaidi *et al.* 2008). Cells lacking *MMS22*, *RTT101*, or *MMS1* showed similarly increased sensitivities to MMS and HU. The importance of the Mms22p-Mms1p module in stabilizing the replisome during replication stress is conserved in both budding yeast and fission yeast (Dovey and Russell 2007; Zaidi *et al.* 2008; Vaisica *et al.* 2011). However, no clear ortholog to *ScRTT101* has been identified in *S. pombe*.

The *C. albicans rtt101* null mutant displayed wild-type colony growth on both solid media and liquid media, which was similar to the *rad57*-null mutant (Figure 6). The *rtt101*-null mutant was sensitive to MMS but not to HU, CPT, or IR (Figure 7; Figure 2A in SC-Met^−^/Cys^−^ medium). Compared with the P*_MET3_-MMS22* single mutant, the P*_MET3_-MMS22*/Δ*rtt101* strain exhibited enhanced sensitivity to MMS, similar sensitivity to CPT and IR, and unchanged sensitivity to HU (Figure 2A; Table 3), suggesting that Rtt101p might work together with Mms22p in the same pathway in response to MMS.

## Discussion

In this study, we identified and characterized a DNA-repair protein, Mms22p, in *C. albicans*. In untreated cells, repression of the *MMS22* gene resulted in elongated and deformed cells. Shut off of the P*_MET3_-MMS22* mutant on solid SC-Met^+^/Cys^+^ medium caused hypersensitivity to the chemical agents MMS and CPT as well as IR. Moreover, after transient exposure to MMS, P*_MET3_-MMS22* mutants were unable to complete mitosis in a timely fashion and showed decreased viability, accumulating with an elongated morphology and arresting in S phase. In *C. albicans*, Mms22p likely participates in the DNA repair pathway that is important for the recovery from S-phase-specific DNA damage caused by MMS, CPT or IR. As well, Mms22p is required for normal cell cycle progression after recovery from replication stress.

In *S. cerevisiae*, Mms22p has been proposed to be a substrate-specific adaptor of a DNA repair−specific Rtt101p-based cullin complex that is stimulated by MMS, works in an Mms1p-dependent manner, and is involved in the processing of stalled replication forks (Zaidi *et al.* 2008). Cullins are a family of proteins that act as scaffolds for the assembly of multisubunit ubiquitin ligases. Protein ubiquitination involves three enzymes: E1, E2, and a ubiquitin ligase E3,which can directly recognize specific substrates to perform different functions (Mellon *et al.* 1987; Dovey and Russell 2007; Daulny and Tansey 2009; Fujii *et al.* 2009). Rtt101p is a cullin-based protein that forms part of an E3 ubiquitin ligase complex required for replication fork progression through DNA lesions and naturally occurring pause sites in yeast (Luke *et al.* 2006). In response to DNA damage, Rtt101p is recruited to chromatin, in a process that depends on the histone H3 lysine-56 acetyltransferase Rtt109p and the BRCA1 C terminus repeat-containing protein Rtt107p (Roberts *et al.* 2008).

Each component of the Rtt101p−Mms1p−Mms22p complex is important for the stable association of the replisome with replication forks during replication stress (Vaisica *et al.* 2011). Moreover, an evolutionary conserved Mms1p−Mms22p module also is required for replication of damaged DNA in fission yeast (Dovey *et al.* 2009). Although no clear ortholog of Mms1p has been identified in *C. albicans*, the Δ*rtt101* mutant displayed similar sensitivities to the chemical agents and IR, as did the P*_MET3_-MMS22* mutant, which suggests that Mms22p and Rtt101p may function in the same pathway in the presence of replication-associated DNA damage and is consistent with the Rtt101p−Mms22p complex (either without Mms1p or with a structurally highly divergent Mms1p) also being essential for the stabilization of the replisome during replication stress in *C. albicans*.

During DNA synthesis, replication forks are exposed to various types of stress. Csm3p, Tof1p, and Mrc1p have been identified as checkpoint-specific mediators in budding yeast, and they have the overlapping role during activation the replication checkpoint (Foss 2001; Osborn and Elledge 2003; Tong *et al.* 2004). Recent studies suggested that Mrc1p was required to maintain the normal rate of replication fork progression, whereas Tof1p was critical for DNA replication forks to pause at diverse chromosomal sites where non-nucleosomal proteins bind very tightly to DNA (Bando *et al.* 2009). Swi1p and Swi3p of *S. pombe*, the homologs of *S. cerevisiae* Tof1p and Csm3p, form a complex and play important roles in the stabilization of stalled replication forks and activation of the DNA replication checkpoint (Noguchi *et al.* 2004). Our study suggests that although Mrc1p and Csm3p are involved in DNA replication and repair in *C. albicans*, Tof1p is apparently not required for these processes. In the absence of exogenous DNA damaging agents, when either *MRC1* or *CSM3* was repressed, the cells exhibited a mitotic delay and were arrested in the G2 phase with a constitutively pseudohyphal morphology. Furthermore, these mutants had increased sensitivity to the agents MMS, HU, CPT, and IR, resulting in reduced viability compared with the wild-type strain; this occurred whether Mms22p was repressed or not. Usually, pseudohyphae and true hyphae emerge in response to cell-cycle arrest in *C. albicans* (Berman 2006). We speculate that the delayed cell cycle in either mutants or cells treated with reagents that alter cell-cycle progression can cause cell elongation in *C. albicans*. This point is consistent with the view that cell polarity during hyphal morphogenesis is regulated by a change in the cell cycle (Ahn *et al.* 1999; Loeb *et al.* 1999). Our results suggest that the mutants in which the replisome components Mrc1p or Csm3p were repressed were unable to recover from DNA damage, supporting an important role for Mrc1p and Csm3p in DNA repair in the fungal pathogen.

Mms22p, together with Mms1p, is indispensable for the stabilization of the *S. cerevisiae* Mrc1p−Csm3p−Tof1p component under conditions of replication stress. The deletion of *MMS22* reduces either Mrc1p or Csm3p localization to stalled replication forks (Dovey and Russell 2007; Vaisica *et al.* 2011). In contrast, *S. pombe* has a negative relationship between Mms22p and Swi1p or Swi3p. The deletion of either *swi1* or *swi3* rescues the phenotypes in the *mms22* mutant (Table 3) (Dovey and Russell 2007). Similarly to the situation in *S. pombe*, in this study, we observed that the repression of either *CSM3* or *MRC1* led to a partial rescue of the sensitivity of the P*_MET3_-MMS22* mutant to MMS, whereas the repression of *MRC1* caused increased the sensitivity to CPT and IR of the P*_MET3_-MMS22* mutant. This finding suggests that Mms22p is required for responding to DNA damage agents in *MRC1* or *CSM3* conditional mutants.

In the budding yeast, Rad51p-mediated HR plays a central role in promoting repair of double-strand breaks generated during replication (Heyer *et al.* 2010; Holthausen *et al.* 2010). HR is initiated at regions of single-strand DNA that become coated by the evolutionarily conserved Rad51p recombinase to form nucleoprotein filaments. These filaments, assisted by Rad52p and Rad55p-Rad57p, facilitate the search for homologous sequences in an intact duplex that acts as a template for repair synthesis (Paques and Haber 1999; Herzberg *et al.* 2006; Wu and Hickson 2006). In this study, the Δ*rad57* mutant was more sensitive to MMS and IR and especially to HU compared with P*_MET3_-MMS22* mutant. The Δ*rad57* mutant was hypersensitive to CPT in the absence of *MMS22*. Our results suggest that Mms22p is only essential for HR induced by CPT. This finding is in contrast to the requirement of Mms22p in budding yeast for HR-mediated repair (Duro *et al.* 2008), or the blockage action of *S. pombe* Mms22p for HR repair pathway (Dovey and Russell 2007).

In conclusion, our results show that although *C. albicans* orthologs of *S. cerevisiae* and *S. pombe* DNA damage repair pathway members are involved in DNA damage repair in the fungal pathogen, the details of their function show distinct characteristics. In the pathogen Mms22p has little role in protecting against HU-mediated damage, whereas Tof1p appears unimportant in response to any damage investigated, in sharp contrast to their importance in these roles in *S. cerevisiae*. Overall, in the pathogen it appears that Mms22p plays a critical role in preserving genome integrity during DNA replication; perhaps Mms22 functions to maintain genomic integrity by HR through coordination of DNA synthesis by interacting with Rtt101p in the rescue of paused replication forks after they confront a block.

## Supplementary Material

Supporting Information
