# Supplementary material for: The Role of Mms22p in DNA Damage Response in Candida albicans
Source: G3 (Bethesda). 2015 Oct 4;5(12):2567–78. doi: 10.1534/g3.115.021840 (PMC4683630; doi:10.1534/g3.115.021840)
Supplement: Supporting Information [file supp_5_12_2567__index.html]

The Role of Mms22p in DNA Damage Response in Candida albicans — Supporting Information 

# The Role of Mms22p in DNA Damage Response in *Candida albicans*

## Supporting Information for Yan *et al.*, 2015

**Files in this Data Supplement:**

- Supporting Information - Figures S1-S4, File S1, and Table S1 (PDF, 2.1 MB)
- Figure S1 - Protein sequence alignment of Mms22p (PDF, 1.4 MB)
- Figure S2 - (A) Strain construction (B) PCR confirmation of disruption of *MMS22* by genomic DNA(PDF, 175 KB)
- Figure S3 - (A) Strain construction (B) PCR confirmation of disruption of *TOF1*, *CSM3*,or *MRC1* by genomic DNA (PDF, 347 KB)
- Figure S4 - (A) Strain construction (B) PCR confirmation of deletion of *RAD57*, or *RTT101* by genomic DNA (PDF, 327 KB)
- File S1 - Supporting Materials and Methods (PDF, 73 KB)
- Table S1 - The oligonucleotides used in this study (PDF, 106 KB)
