# Supplementary material for: The Role of Mms22p in DNA Damage Response in Candida albicans
Source: G3 (Bethesda). 2015 Oct 4;5(12):2567–78. doi: 10.1534/g3.115.021840 (PMC4683630; doi:10.1534/g3.115.021840)
Supplement: Supporting Information [file supp_g3.115.021840_021840SI.pdf]

The role of Mms22p in DNA damage response in *Candida albicans*

Lan Yan[*1], Juan Xiong[*,‡1], Hui Lu[‡], Quan-zhen Lv[*], Qian-yao Ma[§], Pierre Côte[†], Malcolm Whiteway[†2], Yuan-ying Jiang[*2]

[*] Center for New Drug Research, School of Pharmacy, Second Military Medical University, 325 Guohe Road, Shanghai 200433, P. R. China;
[†] Department of Biology, Concordia University, 7141 Sherbrooke Street West, Montreal, Quebec, H4B 1R6, Canada
[‡] Key Laboratory of the Plateau of the Environmental Damage Control, Lanzhou General Hospital of Lanzhou Military Command, No.333 Binhe South Road, Lanzhou, 730050, China.
[§]School of Life Science and Bio-pharmaceutics, Shenyang Pharmaceutical University, Shenyang, Liaoning Province 110016, P.R. China

[1] These authors contributed equally to this work.

[2] Corresponding authors:

Dr. Malcolm Whiteway
Department of Biology, Concordia University, 7141 Sherbrooke Street West, Montreal, Quebec, H4B 1R6, Canada
Email: malcolm.whiteway@concordia.ca; Phone 514-848-2424-3751
Dr. Yuan-ying Jiang
Center for New Drug Research, School of Pharmacy, Second Military Medical University; 325 Guohe Road, Shanghai 200433, P. R. China
Email: 13761571578@163.com; Phone: 086-021-81871357; Fax: 086-021-81871275

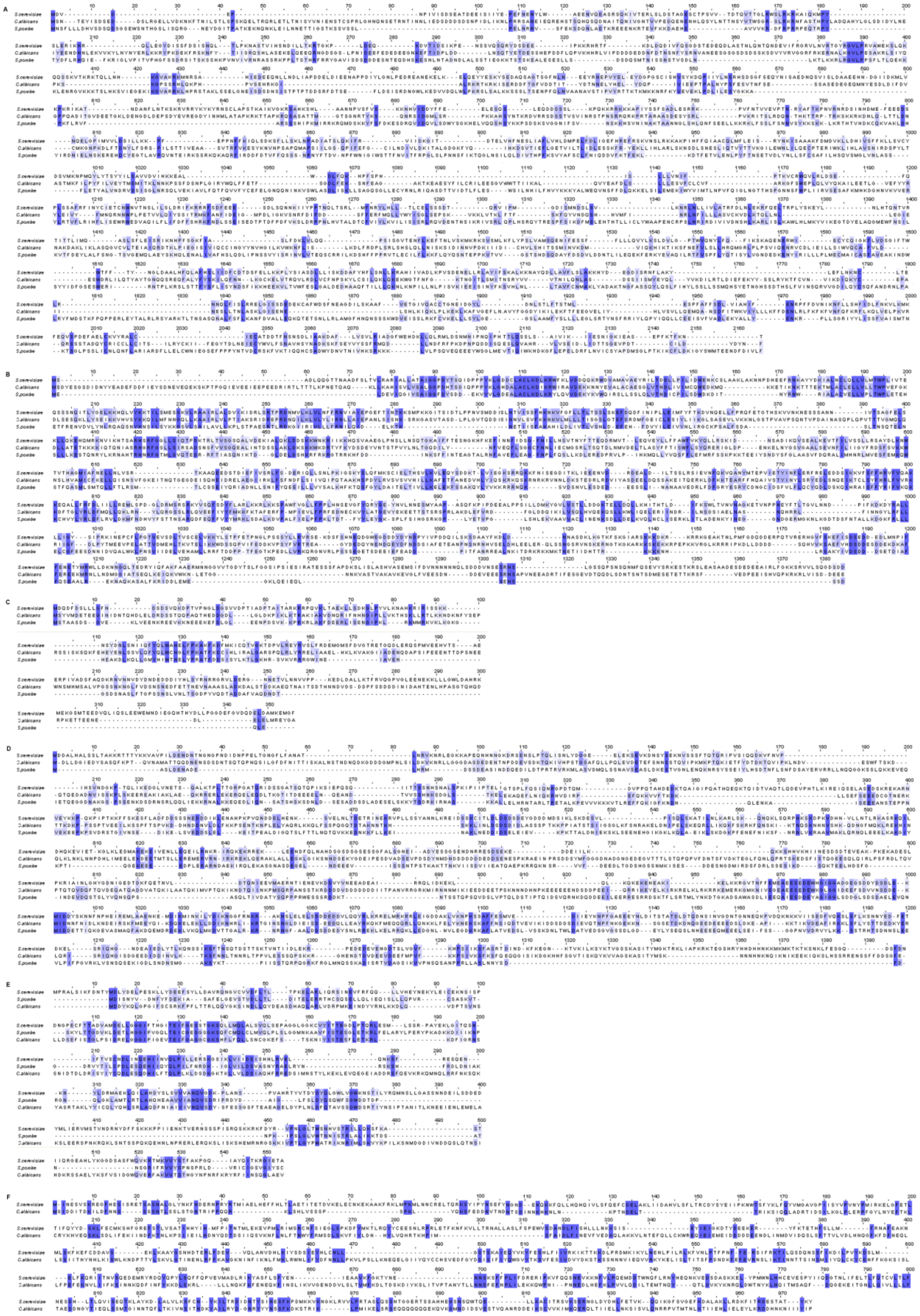

Figure S1. Protein sequence alignment of Mms22p (A), Tof1p (B), Csm3p (C), Mrc1p (D), Rad57p (E), and Rtt101p (F).

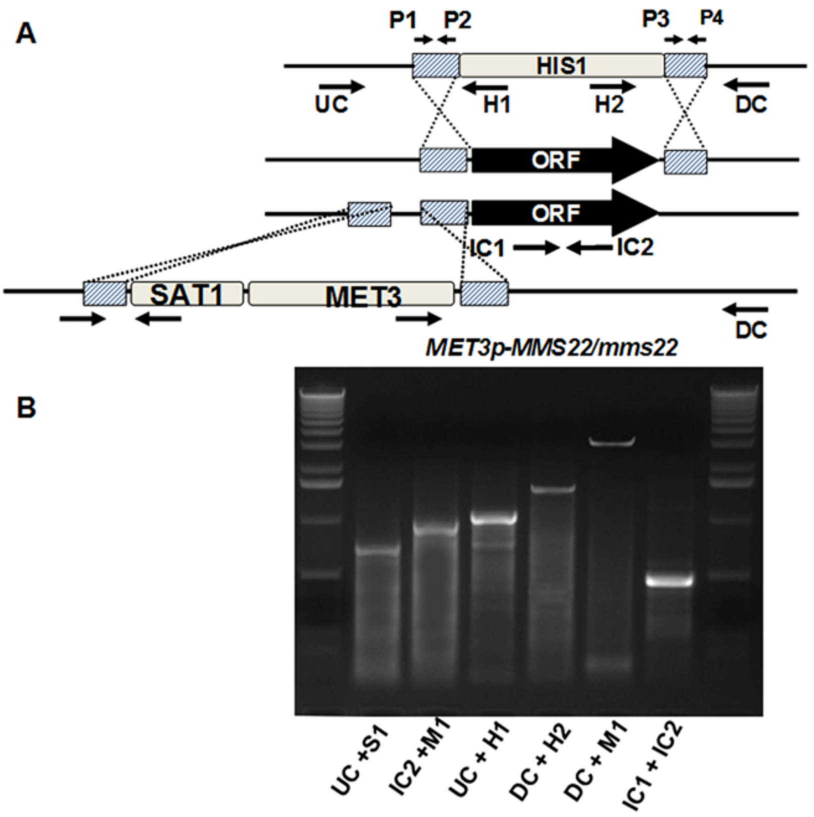

Figure S2. (A) Strain construction. For detailed explanations of the technique, please see the supplementary Materials and Methods. Small arrows represent orientation and approximate position of oligonucleotides (Table S1) used for fusion PCR and confirmation of the disruption. (B) PCR confirmation of disruption of *MMS22* by genomic DNA. The mutant was analyzed by genomic DNA amplified with the oligonucleotides indicated at the bottom of the figure.

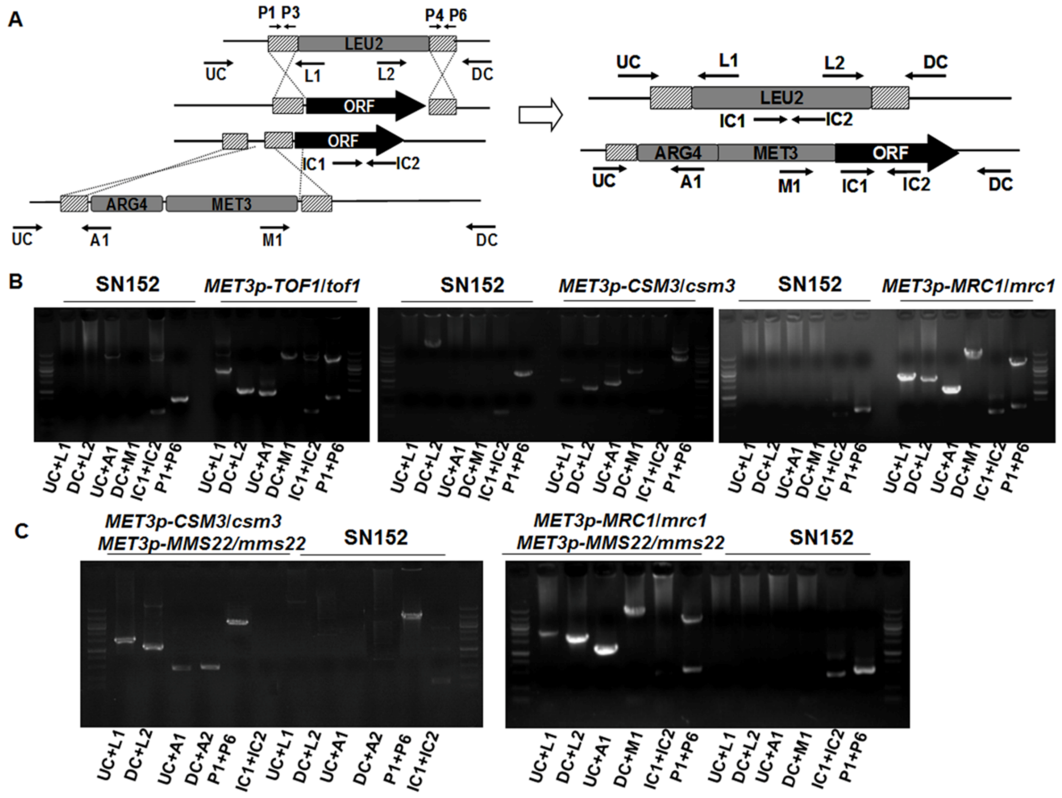

Figure S3. (A) Strain construction. For detailed explanations of the technique, please see supplementary Materials and Methods. Small arrows represent orientation and approximate position of oligonucleotides (Table S1) used for fusion PCR and confirmation of the disruption. (B) PCR confirmation of disruption of *TOF1*, *CSM3*, or *MRC1* by genomic DNA. The mutant was analyzed by genomic DNA amplified with the oligonucleotides indicated at the bottom of the figure. (C) PCR confirmation of construction of P_{MET3}-*MMS22*/P_{MET3}-*MRC1*, P_{MET3}-*MMS22*/P_{MET3}-*CSM3* mutants by genomic DNA.

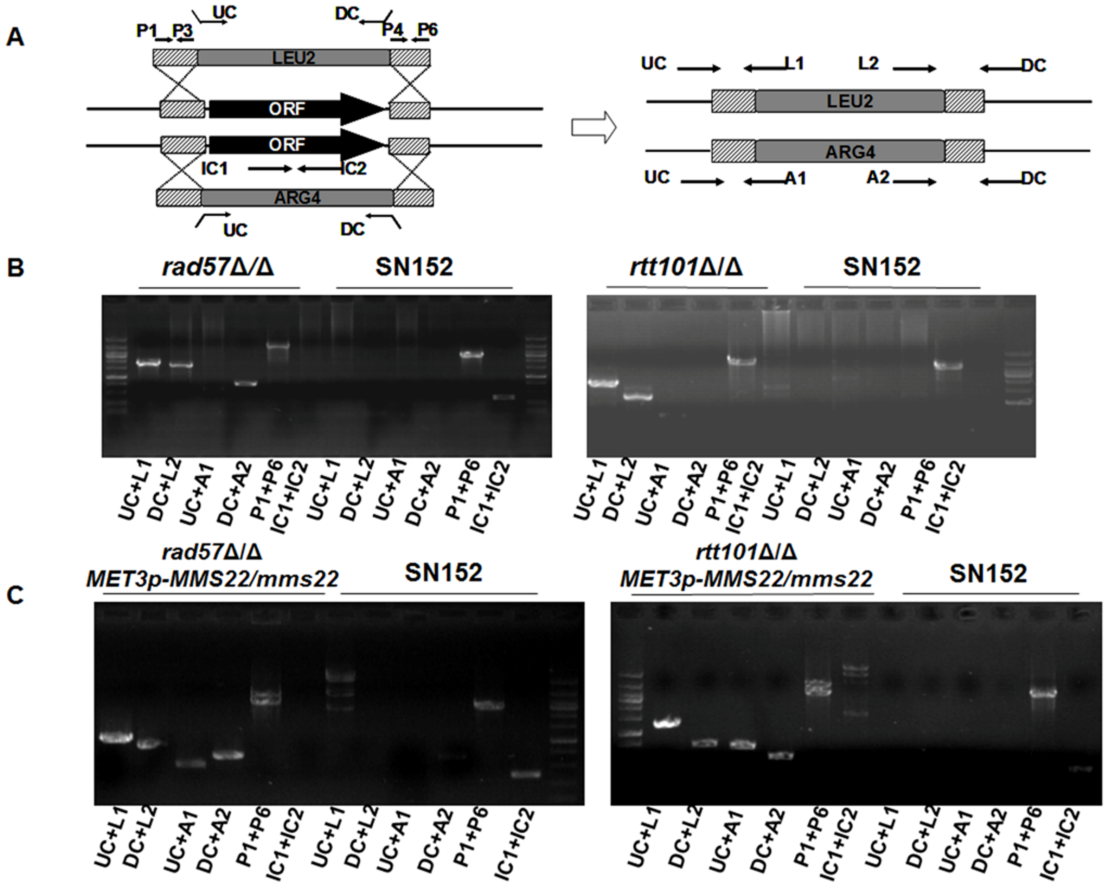

Figure S4. (A) Strain construction. For detailed explanations of the technique, please see supplementary Materials and Methods. Small arrows represent orientation and approximate position of oligonucleotides (Table S1) used for fusion PCR and confirmation of the disruption. (B) PCR confirmation of deletion of *RAD57*, or *RTT101* by genomic DNA. The mutant was analyzed by genomic DNA amplified with the oligonucleotides indicated at the bottom of the figure. (C) PCR confirmation of construction of P_{MET3}-*MMS22*/Δ*rad57* and P_{MET3}-*MMS22*/Δ*rtt101*. All the mutants were analyzed by genomic DNA amplified with the oligonucleotides indicated at the bottom of the figure.

File S1

Supporting Materials and Methods

Strain constructions

CaLY226 (*MET3p-MMS22*/*mms22*::*C.d.HIS1*): The *SAT1-MET3p* cassette from plasmid pFA-SAT1-MET3p was amplified using the primers oLY152 and oLY153 to generate a *SAT1-MET3p-MMS22* cassette with 100 base pairs (bps) of homology to the *MMS22* 5' upstream region and 100 bps of homology to the beginning of the *MMS22* ORF. Primers oLY83 and oLY84 were used to amplify genomic DNA on the 5' side of the *MMS22* gene; primers oLY85 and oLY86 were used to amplify genomic DNA on the 3' side of the *MMS22* gene; primers oLY232 and oLY233 were used to amplify the *C.d.HIS1* sequence from plasmid pSN52. These three fragments were fused to generate *mms22*::*C.d.HIS1* disruption cassette. *C. albicans* SN152 was then transformed with these cassettes to generate strains CaLY8 (*MMS22*/*mms22*::*C.d.HIS1*) and CaLY226 (*MET3p-MMS22*/*mms22*::*C.d.HIS1*). Proper integration of *SAT1-MET3p-MMS22* cassette was verified by genomic PCR using primers oLY87 and oLY300 as well as oLY88 and oLY301. Correct integration of *mms22*::*C.d.HIS1* disruption cassette was confirmed with primers oLY87 plus oLY236 as well as oLY88 plus oLY237. Presence of *MMS22* was verified with primers oLY89 and oLY90.

CaLY337 (*ARG4-MET3p-TOF1*/*tof1*::*C.m.LEU2*): The *ARG4-MET3p* cassette from plasmid pFA-ARG4-MET3p was amplified using the primers oLY312 and oLY313, and then fused with the upstream region (amplified with oLY534 and oLY535) and the beginning of the *TOF1* ORF (amplified with oLY536 and oLY537) to generate an *ARG4-MET3p-TOF1* cassette. Primers oLY160 and oLY161 were used to amplify genomic DNA on the 5' side of the *TOF1* gene; primers oLY162 and oLY163 were used to amplify genomic DNA on the 3' side of the *TOF1* gene; primers oLY232 and oLY233 were used to amplify the *C.m.LEU2* sequence from plasmid pSN40. These three fragments were fused to generate *tof1*::*C.m.LEU2* disruption cassette. *C. albicans* SN152 was then transformed with these cassettes to generate strains CaLY219 (*TOF1*/*tof1*::*C.m.LEU2*) and CaLY337 (*ARG4-MET3p-TOF1*/*tof1*::*C.m.LEU2*). Proper integration of *ARG4-MET3p-TOF1* cassette was verified by genomic PCR using primers oLY461 and oLY538 as well as oLY462 and oLY301. Proper integration of *tof1*::*C.m.LEU2* disruption cassette was verified with primers oLY461 and oLY366 as well as oLY462 and oLY367. Presence of *TOF1* was verified with primers oLY463 and oLY464.

CaLY249 (*ARG4-MET3p-CSM3*/*csm3*::*C.m.LEU2*): The *ARG4-MET3p* cassette from plasmid pFA-ARG4-MET3p was amplified using the primers oLY312 and oLY313, and then fused with the upstream region (amplified with oLY543 and oLY544) and the beginning of the *CSM3* ORF (amplified with oLY545 and oLY546) to generate a *ARG4-MET3p-CSM3* cassette. Primers oLY465 and oLY466 were used to amplify genomic DNA on the 5' side of the *CSM3* gene; primers oLY467 and oLY468 were used to amplify genomic DNA on the 3' side of the *CSM3* gene; primers oLY232 and oLY233 were used to amplify the *C.m.LEU2* sequence from plasmid pSN40. These three fragments were fused to generate *csm3*::*C.m.LEU2* disruption cassette. *C. albicans* SN152 was then transformed with these cassettes to generate strains CaLY220 (*CSM3*/*csm3*::*C.m.LEU2*) and CaLY249 (*ARG4-MET3p-CSM3*/*csm3*::*C.m.LEU2*). Proper integration of *ARG4-MET3p-CSM3* cassette was verified by genomic PCR using primers oLY469 and oLY538 as well as oLY470 and oLY301. Proper integration of *csm3*::*C.m.LEU2* disruption cassette was verified with primers oLY469 and oLY366 as well as oLY470 and oLY367. Presence of *CSM3* was verified with primers oLY471 and oLY472.

CaLY316 (*ARG4-MET3p-MRC1*/*mrc1*::*C.m.LEU2*): The *ARG4-MET3p* cassette from plasmid pFA-ARG4-MET3p was amplified using the primers oLY312 and oLY313 and then fused with the upstream region (amplified with oLY539 and oLY540) and the beginning of the *MRC1* ORF (amplified with oLY541 and oLY542) to generate a *ARG4-MET3p-MRC1* cassette. Primers oLY174 and oLY175 were used to amplify genomic DNA on the 5' side of the *MRC1* gene; primers oLY176 and oLY177 were used to amplify genomic DNA on the 3' side of the *MRC1* gene; primers oLY232 and oLY233 were used to amplify the *C.m.LEU2* sequence from plasmid pSN40. These three fragments were fused to generate *mrc1*::*C.m.LEU2* disruption cassette. *C. albicans* SN152 was then transformed with these cassettes to generate strains CaLY222 (*MRC1*/*mrc1*::*C.m.LEU2*) and CaLY316 (*ARG4-MET3p-MRC1*/*mrc1*::*C.m.LEU2*). Proper integration of *ARG4-MET3p-MRC1* cassette was verified by genomic PCR using primers oLY477 and oLY538 as well as oLY478 and oLY301. Proper integration of *mrc1*::*C.m.LEU2* disruption cassette was verified with primers oLY477 and oLY366 as well as oLY478 and oLY367. Presence of *MRC1* was verified with primers oLY479 and oLY480.

CaLY235 (*rad57*::*C.m.LEU2*/*rad57*::*C.d.ARG4*): Primers oLY481 and oLY482 were used to amplify genomic DNA on the 5' side of the *RAD57* gene; primers oLY483 and oLY484 were used to amplify genomic DNA on the 3' side of the *RAD57* gene; primers oLY232 and oLY233 were used to amplify the *C.m.LEU2* and *C.d.ARG4* sequence from plasmid pSN40 and pSN69. These fragments were fused to generate

rad57::*C.m.LEU2* and *rad57*::*C.d.ARG4* disruption cassettes. *C. albicans* SN152 was then transformed with these cassettes to generate strains CaLY223 (*RAD57*/*rad57*::*C.m.LEU2*) and CaLY235 (*rad57*::*C.m.LEU2*/*rad57*::*C.d.ARG4*). Proper integration of *rad57*::*C.m.LEU2* cassette was verified by genomic PCR using primers oLY485 and oLY366 as well as oLY486 and oLY367. Proper integration of *rad57*::*C.d.ARG4* disruption cassette was verified with primers oLY485 and oLY238 as well as oLY486 and oLY239. Presence of *RAD57* was verified with primers oLY487 and oLY488.

CaLY236 (*rtt101*::*C.m.LEU2*/*rtt101*::*C.d.ARG4*): Primers oLY489 and oLY490were used to amplify genomic DNA on the 5' side of the *RTT101* gene; primers oLY491 and oLY492 were used to amplify genomic DNA on the 3' side of the *RTT101* gene; primers oLY232 and oLY233 were used to amplify the *C.m.LEU2* and *C.d.ARG4* sequence from plasmid pSN40 and pSN69. These fragments were fused to generate *rtt101*::*C.m.LEU2* and *rtt101*::*C.d.ARG4* disruption cassettes. *C. albicans* SN152 was then transformed with these cassettes to generate strains CaLY224 (*Rtt101*/*rtt101*::*C.m.LEU2*) and CaLY236 (*rtt101*::*C.m.LEU2*/*rtt101*::*C.d.ARG4*). Proper integration of *rtt101*::*C.m.LEU2* cassette was verified by genomic PCR using primers oLY493 and oLY366 as well as oLY494 and oLY367. Proper integration of *rtt101*::*C.d.ARG4* disruption cassette was verified with primers oLY493 and oLY238 as well as oLY494 and oLY239. Presence of *Rtt101* was verified with primers oLY495 and oLY496.

CaLY251 (*MET3p-MMS22*/*mms22*::*C.d.HIS1 ARG4-MET3p-MRC1*/*mrc1*::*C.m.LEU2*): *C. albicans* CaLY226 was transformed with *mrc1*::*C.m.LEU2* and *ARG4-MET3p-MRC1* cassettes to generate strains CaLY228 (*MET3p-MMS22*/*mms22*::*C.d.HIS1 MRC1*/*mrc1*::*C.m.LEU2*) and CaLY251 (*MET3p-MMS22*/*mms22*::*C.d.HIS1 ARG4-MET3p-MRC1*/*mrc1*::*C.m.LEU2*).

CaLY246 (*MET3p-MMS22*/*mms22*::*C.d.HIS1 ARG4-MET3p-CSM3*/*csm3*::*C.m.LEU2*): *C. albicans* CaLY226 was transformed with *csm3*::*C.m.LEU2* and *ARG4-MET3p-CSM3* cassettes to generate strains CaLY234 (*MET3p-MMS22*/*mms22*::*C.d.HIS1 CSM3*/*csm3*::*C.m.LEU2*) and CaLY246 (*MET3p-MMS22*/*mms22*::*C.d.HIS1 ARG4-MET3p-CSM3*/*csm3*::*C.m.LEU2*).

CaLY242 (*MET3p-MMS22*/*mms22*::*C.d.HIS1 rad57*::*C.m.LEU2*/*rad57*::*C.d.ARG4*): *C. albicans* CaLY226 was transformed with *rad57*::*C.m.LEU2* and *rad57*::*C.d.ARG4* cassettes to generate strains CaLY238 (*MET3p-MMS22*/*mms22*::*C.d.HIS1 RAD57*/*rad57*::*C.m.LEU2*) and CaLY242 (*MET3p-MMS22*/*mms22*::*C.d.HIS1*

rad57::*C.m.LEU2*/*rad57*::*C.d.ARG4*).

CaLY244 (*MET3p-MMS22*/*mms22*::*C.d.HIS1 rtt101*::*C.m.LEU2*/*rtt101*::*C.d.ARG4*): *C. albicans* CaLY226 was transformed with *rtt101*::*C.m.LEU2* and *rtt101*::*C.d.ARG4* cassettes to generate strains CaLY240 (*MET3p-MMS22*/*mms22*::*C.d.HIS1 Rtt101*/*rtt101*::*C.m.LEU2*) and CaLY244 (*MET3p-MMS22*/*mms22*::*C.d.HIS1 rtt101*::*C.m.LEU2*/*rtt101*::*C.d.ARG4*).

Table S1 A supporting table. The oligonucleotides used in this study.

Strains	Primer	Primer sequence
CaLY8	P1 (oLY83)	CAAGACCATTTACAAGCAATCC
	P3 (oLY84)	cacggcgcgcctagcagcggTTGAAAGTGGGAACAAGGTTAG
	P4(oLY85)	gtcagcggccgcatccctgcCGATTCCAATTTGTCTTTGGC
	P6(oLY86)	AACAAGAACCAGTCCCACC
	UC(oLY87)	TGGTAAACTTATTCGTGCTGG
	DC(oLY88)	ACAGCAGAAGACTTGAAAGAAC
	IC1(oLY89)	ACGACGATTCAGATTCAAACC
	IC2(oLY90)	GCTCTTCTTGAAGCTCTTTTTC
CaLY226	oLY152	CGCTTGGGCGACACTGTGGTGGCAAAGTAGTGCGACGTAGGTGCAAGTCTAAGACGAAGAAAAACTAGGGAAAGGCAAACGCGTCCAGCAATATTATTTTgaagcttcgtacgctgcaggtc
	oLY153	GATTATTATGAAAAATGCTAAAGTAGTGAATGAAACTGTGTTATATCTTTTAATATTATCATTAAGTTATGGTTCTATTTATATTTGAAAGTGGGAACAAcatgttttctggggagggtatttac
	UC(oLY87)	TGGTAAACTTATTCGTGCTGG
	DC(oLY88)	ACAGCAGAAGACTTGAAAGAAC
	IC1(oLY89)	ACGACGATTCAGATTCAAACC
	IC2(oLY90)	GCTCTTCTTGAAGCTCTTTTTC
CaLY219	P1(oLY160)	TCGAGGATAGACCGTGAACC
	P3(oLY161)	cacggcgcgcctagcagcggTTGAGAAGGCACAGCAACAG
	P4(oLY162)	gtcagcggccgcatccctgcAAAAGAAACGCCCTGAACCT
	P6(oLY163)	CTTTCACAGCTTTTGCCACA
	UC(oLY461)	CAAGTTGGCTGGTGAAGTGA
	DC(oLY462)	GGCGGAGACCATTGTGTAAT
	IC1(oLY463)	GATCGAGAGTTGGCAGAAGG
	IC2(oLY464)	GCTTGATGGAAAAACCTTGC
CaLY337	P1(oLY534)	GAAACTTGGCTTGGGTCAAT
	P3(oLY535)	cacggcgcgcctagcagcggAAAATCACCACGAACCCATC
	P4(oLY536)	gtcagcggccgcatccctgcATGAGTGATTATGAATCAGG
	P6(oLY537)	AGGCGGTGGTTGAATATCTG
	UC(oLY461)	CAAGTTGGCTGGTGAAGTGA
	DC(oLY462)	GGCGGAGACCATTGTGTAAT
	IC1(oLY463)	GATCGAGAGTTGGCAGAAGG
	IC2(oLY464)	GCTTGATGGAAAAACCTTGC
CaLY220	P1(oLY465)	GGAGAGGAATTCTCCAGCAA

CaLY234	P3(oLY466)	cacggcgcgcctagcagcggTGGAACGCGTTTATTATGGTC
	P4(oLY467)	gtcagcggccgcatccctgcTGAGAGAGTACGGCGCATAA
	P6(oLY468)	GGACAAGAGGTTTTCGGATG
	UC(oLY469)	CGTCAATAGGTGGGCTGTTT
	DC(oLY470)	TGACCAAACGCAAAAACGTA
	IC1(oLY471)	TGTGGTGTCATGGCTTGTTT
	IC2(oLY472)	CTGGGATACTTGGTGCGTCT
CaLY249	P1(oLY543)	TCCTGTTATGCTTGCTTGTGA
CaLY246	P3(oLY544)	cacggcgcgcctagcagcggCGTAATGGGAAGACGGAAAA
	P4(oLY545)	gtcagcggccgcatccctgcATGTCATATGTGATGGACGA
	P6(oLY546)	AACAAGCCATGACACCACAA
	UC(oLY469)	CGTCAATAGGTGGGCTGTTT
	DC(oLY470)	TGACCAAACGCAAAAACGTA
	IC1(oLY471)	TGTGGTGTCATGGCTTGTTT
	IC2(oLY472)	CTGGGATACTTGGTGCGTCT
CaLY222	P1(oLY174)	AGATTGTTAGGAGGCGGTGA
CaLY228	P3(oLY175)	cacggcgcgcctagcagcggTTTCACGACGTTTTTGTTCG
	P4(oLY176)	gtcagcggccgcatccctgcAACCAAGGTGAAGAAGACGAAG
	P6(oLY177)	ATTTTCATGGCCCCTCTTTT
	UC(oLY178)	TGGCCATCAGGAAAGTTGA
	DC(oLY179)	ACTGCTGGGAACCGATAATG
	IC1(oLY180)	TGGCAATGGTGAAGATGAAG
	IC2(oLY181)	TTTTACGACCACGACGAACA
CaLY316	P1(oLY539)	ACAGTGATTGTCGTTTATTCAAGAG
CaLY251	P3(oLY540)	cacggcgcgcctagcagcggATGCGAGCATCCCAATTCTA
	P4(oLY541)	gtcagcggccgcatccctgcATGGATTTGTTAGATGGGAT
	P6(oLY542)	CATCATCACCCTTGTCTTGG
	UC(oLY178)	TGGCCATCAGGAAAGTTGA
	DC(oLY179)	ACTGCTGGGAACCGATAATG
	IC1(oLY180)	TGGCAATGGTGAAGATGAAG
	IC2(oLY181)	TTTTACGACCACGACGAACA
CaLY223	P1(oLY481)	ATGTTTGGGAGACGTGGTTG
CaLY235	P3(oLY482)	cacggcgcgcctagcagcggGTCTCGTTCACACGAAAGCA
CaLY238	P4(oLY483)	gtcagcggccgcatccctgcCAACCAACCAACGTGCTAGA
CaLY242	P6(oLY484)	AAATTCTCTCGCAGTGCAGTC
	UC(oLY485)	TCGTTTGAAAGACCACCACA
	DC(oLY486)	TCGTTTTTCCCTCTCGATTG
	IC1(oLY487)	TGGATTCAGACAAGGGGAAG
	IC2(oLY488)	AATCAAGTTCTCCCGCCTCT

CaLY224	P1(oLY489)	GGGTGTGAATCGAATGTATGAA
CaLY236	P3(oLY490)	cacggcgcgcctagcagcggGATTGATGGGAAATGGGTTG
CaLY240	P4(oLY491)	gtcagcggccgcatccctgcTCGACGTCCAGTAACTATGACAA
CaLY244	P6(oLY492)	TTGGTTTTCTGGGGAGCTG
	UC(oLY493)	GCCATTTTCCCCTTGTTTTT
	DC(oLY494)	TTTGGTTTTCTGGGGAGCTG
	IC1(oLY495)	ATCACTGTACCAACGGCAAA
	IC2(oLY496)	CCCATTGTCATCTTCTGCTG
	oLY232	ccgctgctaggcgcgccgtgAGCTCGGATCCACTAGTAACG
	oLY233	gcagggatgcggccgctgacGCCAGTGTGATGGATATCTGC
	oLY236	CAAACACAACTGCACAATCTGGC
	oLY237	GATACGTTGGTGGTTCAGTTGAGG
	oLY238	TTACAAGTATGAAAGGAGGGG
	oLY239	CTTCAACCTTTCAAACGATGC
	oLY300	GCACACACTACTTAATATACACAGC
	oLY301	TCAAGTATACGTAATCTCCCC
	oLY312	ccgctgctaggcgcgccgtgGaagcttcgtacgctgcaggtc
	oLY313	gcagggatgcggccgctgacCatgttttctggggagggtatttac
	oLY538	TCATGCCATTCTTGTCTGAT
	oLY366	GCACGCCGTTACAGGAGTTA
	oLY367	GAAGTTGGTGACGCGATTGT
