# Supplementary figures and images for: The Role of Mms22p in DNA Damage Response in Candida albicans
Source: G3 (Bethesda). 2015 Oct 4;5(12):2567–78. doi: 10.1534/g3.115.021840 (PMC4683630; doi:10.1534/g3.115.021840)

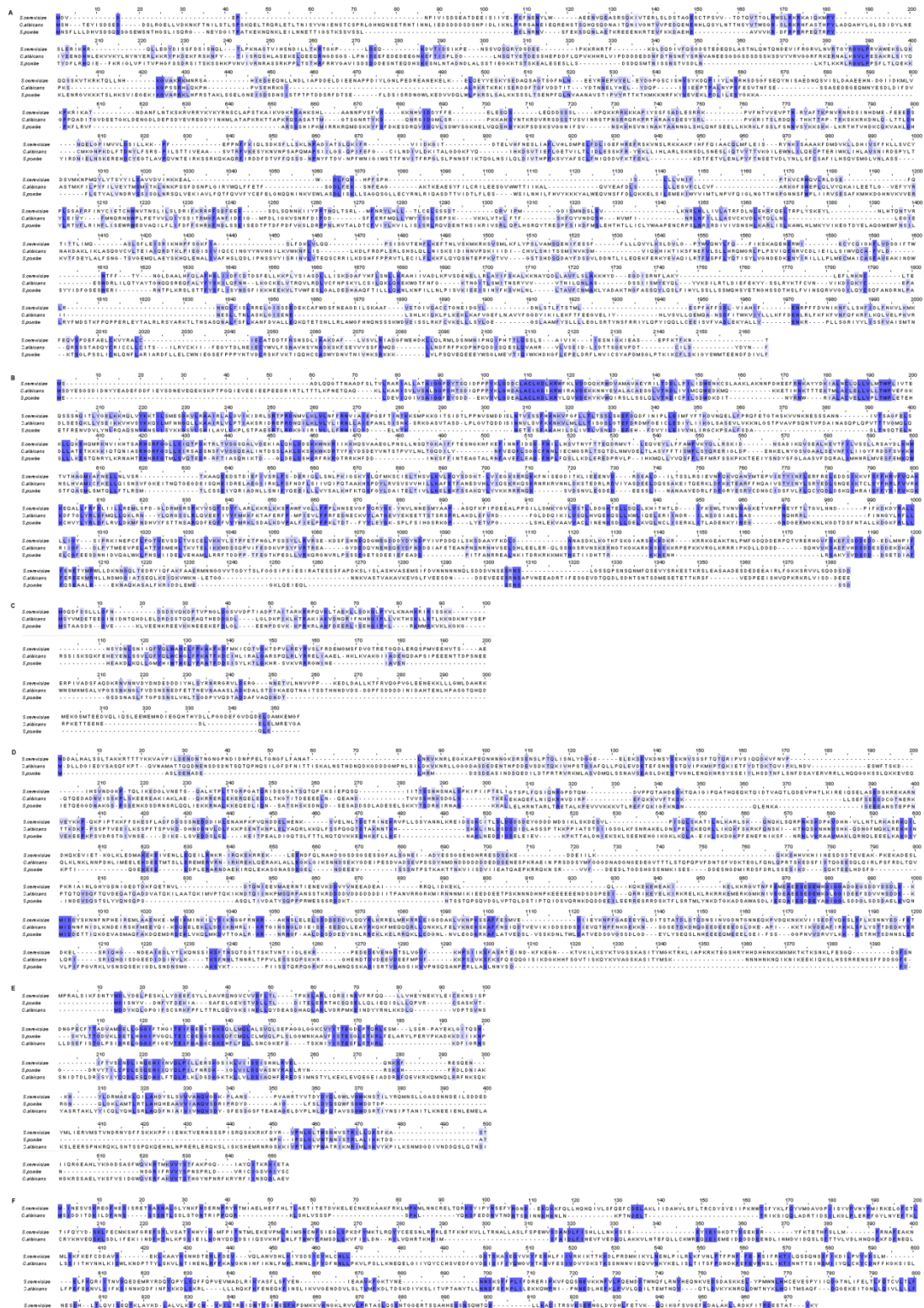

**Figure S1.** Protein sequence alignment of Mms22p (A), Tof1p (B), Csm3p (C), Mrc1p (D), Rad57p (E), and Rtt101p (F).

Supplement: Supporting Information [file supp_g3.115.021840_FigureS1.pdf]
