# Supplementary material for: The Role of Mms22p in DNA Damage Response in Candida albicans
Source: G3 (Bethesda). 2015 Oct 4;5(12):2567–78. doi: 10.1534/g3.115.021840 (PMC4683630; doi:10.1534/g3.115.021840)
Supplement: Supporting Information [file supp_g3.115.021840_FigureS2.pdf]

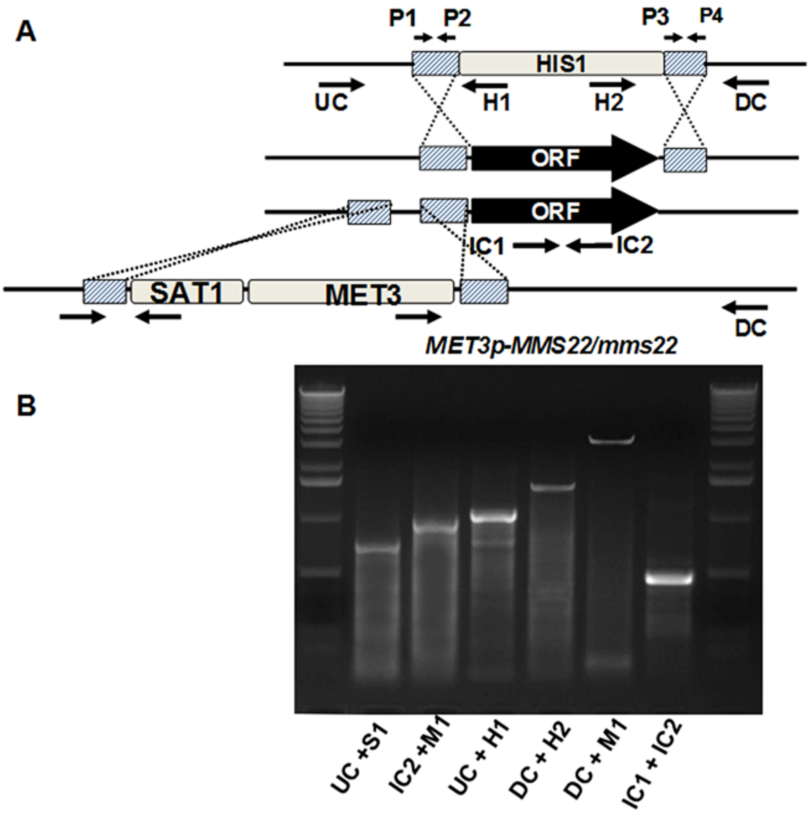

Figure S2. (A) Strain construction. For detailed explanations of the technique, please see the supplementary Materials and Methods. Small arrows represent orientation and approximate position of oligonucleotides (Table S1) used for fusion PCR and confirmation of the disruption. (B) PCR confirmation of disruption of *MMS22* by genomic DNA. The mutant was analyzed by genomic DNA amplified with the oligonucleotides indicated at the bottom of the figure.
