# Supplementary material for: The Role of Mms22p in DNA Damage Response in Candida albicans
Source: G3 (Bethesda). 2015 Oct 4;5(12):2567–78. doi: 10.1534/g3.115.021840 (PMC4683630; doi:10.1534/g3.115.021840)
Supplement: Supporting Information [file supp_g3.115.021840_FigureS4.pdf]

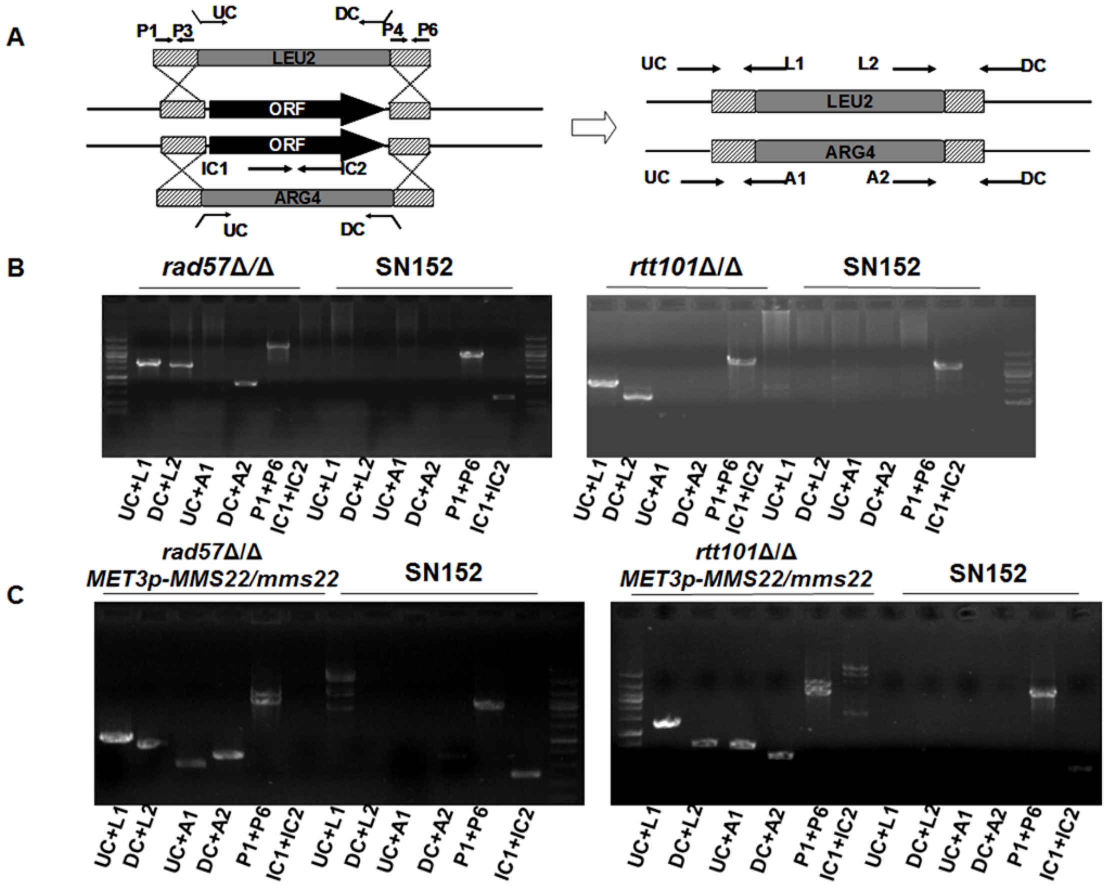

Figure S4. (A) Strain construction. For detailed explanations of the technique, please see supplementary Materials and Methods. Small arrows represent orientation and approximate position of oligonucleotides (Table S1) used for fusion PCR and confirmation of the disruption. (B) PCR confirmation of deletion of *RAD57*, or *RTT101* by genomic DNA. The mutant was analyzed by genomic DNA amplified with the oligonucleotides indicated at the bottom of the figure. (C) PCR confirmation of construction of P_{MET3}-*MMS22*/Δ*rad57* and P_{MET3}-*MMS22*/Δ*rtt101*. All the mutants were analyzed by genomic DNA amplified with the oligonucleotides indicated at the bottom of the figure.
