# Supplementary material for: The Role of Mms22p in DNA Damage Response in Candida albicans
Source: G3 (Bethesda). 2015 Oct 4;5(12):2567–78. doi: 10.1534/g3.115.021840 (PMC4683630; doi:10.1534/g3.115.021840)
Supplement: Supporting Information [file supp_g3.115.021840_FileS1.pdf]

File S1

Supporting Materials and Methods

Strain constructions

CaLY226 (*MET3p-MMS22*/*mms22*::*C.d.HIS1*): The *SAT1-MET3p* cassette from plasmid pFA-SAT1-MET3p was amplified using the primers oLY152 and oLY153 to generate a *SAT1-MET3p-MMS22* cassette with 100 base pairs (bps) of homology to the *MMS22* 5' upstream region and 100 bps of homology to the beginning of the *MMS22* ORF. Primers oLY83 and oLY84 were used to amplify genomic DNA on the 5' side of the *MMS22* gene; primers oLY85 and oLY86 were used to amplify genomic DNA on the 3' side of the *MMS22* gene; primers oLY232 and oLY233 were used to amplify the *C.d.HIS1* sequence from plasmid pSN52. These three fragments were fused to generate *mms22*::*C.d.HIS1* disruption cassette. *C. albicans* SN152 was then transformed with these cassettes to generate strains CaLY8 (*MMS22*/*mms22*::*C.d.HIS1*) and CaLY226 (*MET3p-MMS22*/*mms22*::*C.d.HIS1*). Proper integration of *SAT1-MET3p-MMS22* cassette was verified by genomic PCR using primers oLY87 and oLY300 as well as oLY88 and oLY301. Correct integration of *mms22*::*C.d.HIS1* disruption cassette was confirmed with primers oLY87 plus oLY236 as well as oLY88 plus oLY237. Presence of *MMS22* was verified with primers oLY89 and oLY90.

CaLY337 (*ARG4-MET3p-TOF1*/*tof1*::*C.m.LEU2*): The *ARG4-MET3p* cassette from plasmid pFA-ARG4-MET3p was amplified using the primers oLY312 and oLY313, and then fused with the upstream region (amplified with oLY534 and oLY535) and the beginning of the *TOF1* ORF (amplified with oLY536 and oLY537) to generate an *ARG4-MET3p-TOF1* cassette. Primers oLY160 and oLY161 were used to amplify genomic DNA on the 5' side of the *TOF1* gene; primers oLY162 and oLY163 were used to amplify genomic DNA on the 3' side of the *TOF1* gene; primers oLY232 and oLY233 were used to amplify the *C.m.LEU2* sequence from plasmid pSN40. These three fragments were fused to generate *tof1*::*C.m.LEU2* disruption cassette. *C. albicans* SN152 was then transformed with these cassettes to generate strains CaLY219 (*TOF1*/*tof1*::*C.m.LEU2*) and CaLY337 (*ARG4-MET3p-TOF1*/*tof1*::*C.m.LEU2*). Proper integration of *ARG4-MET3p-TOF1* cassette was verified by genomic PCR using primers oLY461 and oLY538 as well as oLY462 and oLY301. Proper integration of *tof1*::*C.m.LEU2* disruption cassette was verified with primers oLY461 and oLY366 as well as oLY462 and oLY367. Presence of *TOF1* was verified with primers oLY463 and oLY464.

CaLY249 (*ARG4-MET3p-CSM3*/*csm3*::*C.m.LEU2*): The *ARG4-MET3p* cassette from plasmid pFA-ARG4-MET3p was amplified using the primers oLY312 and oLY313, and then fused with the upstream region (amplified with oLY543 and oLY544) and the beginning of the *CSM3* ORF (amplified with oLY545 and oLY546) to generate a *ARG4-MET3p-CSM3* cassette. Primers oLY465 and oLY466 were used to amplify genomic DNA on the 5' side of the *CSM3* gene; primers oLY467 and oLY468 were used to amplify genomic DNA on the 3' side of the *CSM3* gene; primers oLY232 and oLY233 were used to amplify the *C.m.LEU2* sequence from plasmid pSN40. These three fragments were fused to generate *csm3*::*C.m.LEU2* disruption cassette. *C. albicans* SN152 was then transformed with these cassettes to generate strains CaLY220 (*CSM3*/*csm3*::*C.m.LEU2*) and CaLY249 (*ARG4-MET3p-CSM3*/*csm3*::*C.m.LEU2*). Proper integration of *ARG4-MET3p-CSM3* cassette was verified by genomic PCR using primers oLY469 and oLY538 as well as oLY470 and oLY301. Proper integration of *csm3*::*C.m.LEU2* disruption cassette was verified with primers oLY469 and oLY366 as well as oLY470 and oLY367. Presence of *CSM3* was verified with primers oLY471 and oLY472.

CaLY316 (*ARG4-MET3p-MRC1*/*mrc1*::*C.m.LEU2*): The *ARG4-MET3p* cassette from plasmid pFA-ARG4-MET3p was amplified using the primers oLY312 and oLY313 and then fused with the upstream region (amplified with oLY539 and oLY540) and the beginning of the *MRC1* ORF (amplified with oLY541 and oLY542) to generate a *ARG4-MET3p-MRC1* cassette. Primers oLY174 and oLY175 were used to amplify genomic DNA on the 5' side of the *MRC1* gene; primers oLY176 and oLY177 were used to amplify genomic DNA on the 3' side of the *MRC1* gene; primers oLY232 and oLY233 were used to amplify the *C.m.LEU2* sequence from plasmid pSN40. These three fragments were fused to generate *mrc1*::*C.m.LEU2* disruption cassette. *C. albicans* SN152 was then transformed with these cassettes to generate strains CaLY222 (*MRC1*/*mrc1*::*C.m.LEU2*) and CaLY316 (*ARG4-MET3p-MRC1*/*mrc1*::*C.m.LEU2*). Proper integration of *ARG4-MET3p-MRC1* cassette was verified by genomic PCR using primers oLY477 and oLY538 as well as oLY478 and oLY301. Proper integration of *mrc1*::*C.m.LEU2* disruption cassette was verified with primers oLY477 and oLY366 as well as oLY478 and oLY367. Presence of *MRC1* was verified with primers oLY479 and oLY480.

CaLY235 (*rad57*::*C.m.LEU2*/*rad57*::*C.d.ARG4*): Primers oLY481 and oLY482 were used to amplify genomic DNA on the 5' side of the *RAD57* gene; primers oLY483 and oLY484 were used to amplify genomic DNA on the 3' side of the *RAD57* gene; primers oLY232 and oLY233 were used to amplify the *C.m.LEU2* and *C.d.ARG4* sequence from plasmid pSN40 and pSN69. These fragments were fused to generate

rad57::*C.m.LEU2* and *rad57*::*C.d.ARG4* disruption cassettes. *C. albicans* SN152 was then transformed with these cassettes to generate strains CaLY223 (*RAD57*/*rad57*::*C.m.LEU2*) and CaLY235 (*rad57*::*C.m.LEU2*/*rad57*::*C.d.ARG4*). Proper integration of *rad57*::*C.m.LEU2* cassette was verified by genomic PCR using primers oLY485 and oLY366 as well as oLY486 and oLY367. Proper integration of *rad57*::*C.d.ARG4* disruption cassette was verified with primers oLY485 and oLY238 as well as oLY486 and oLY239. Presence of *RAD57* was verified with primers oLY487 and oLY488.

CaLY236 (*rtt101*::*C.m.LEU2*/*rtt101*::*C.d.ARG4*): Primers oLY489 and oLY490were used to amplify genomic DNA on the 5' side of the *RTT101* gene; primers oLY491 and oLY492 were used to amplify genomic DNA on the 3' side of the *RTT101* gene; primers oLY232 and oLY233 were used to amplify the *C.m.LEU2* and *C.d.ARG4* sequence from plasmid pSN40 and pSN69. These fragments were fused to generate *rtt101*::*C.m.LEU2* and *rtt101*::*C.d.ARG4* disruption cassettes. *C. albicans* SN152 was then transformed with these cassettes to generate strains CaLY224 (*Rtt101*/*rtt101*::*C.m.LEU2*) and CaLY236 (*rtt101*::*C.m.LEU2*/*rtt101*::*C.d.ARG4*). Proper integration of *rtt101*::*C.m.LEU2* cassette was verified by genomic PCR using primers oLY493 and oLY366 as well as oLY494 and oLY367. Proper integration of *rtt101*::*C.d.ARG4* disruption cassette was verified with primers oLY493 and oLY238 as well as oLY494 and oLY239. Presence of *Rtt101* was verified with primers oLY495 and oLY496.

CaLY251 (*MET3p-MMS22*/*mms22*::*C.d.HIS1* *ARG4-MET3p-MRC1*/*mrc1*::*C.m.LEU2*): *C. albicans* CaLY226 was transformed with *mrc1*::*C.m.LEU2* and *ARG4-MET3p-MRC1* cassettes to generate strains CaLY228 (*MET3p-MMS22*/*mms22*::*C.d.HIS1* *MRC1*/*mrc1*::*C.m.LEU2*) and CaLY251 (*MET3p-MMS22*/*mms22*::*C.d.HIS1* *ARG4-MET3p-MRC1*/*mrc1*::*C.m.LEU2*).

CaLY246 (*MET3p-MMS22*/*mms22*::*C.d.HIS1* *ARG4-MET3p-CSM3*/*csm3*::*C.m.LEU2*): *C. albicans* CaLY226 was transformed with *csm3*::*C.m.LEU2* and *ARG4-MET3p-CSM3* cassettes to generate strains CaLY234 (*MET3p-MMS22*/*mms22*::*C.d.HIS1* *CSM3*/*csm3*::*C.m.LEU2*) and CaLY246 (*MET3p-MMS22*/*mms22*::*C.d.HIS1* *ARG4-MET3p-CSM3*/*csm3*::*C.m.LEU2*).

CaLY242 (*MET3p-MMS22*/*mms22*::*C.d.HIS1* *rad57*::*C.m.LEU2*/*rad57*::*C.d.ARG4*): *C. albicans* CaLY226 was transformed with *rad57*::*C.m.LEU2* and *rad57*::*C.d.ARG4* cassettes to generate strains CaLY238 (*MET3p-MMS22*/*mms22*::*C.d.HIS1* *RAD57*/*rad57*::*C.m.LEU2*) and CaLY242 (*MET3p-MMS22*/*mms22*::*C.d.HIS1*

rad57::*C.m.LEU2*/*rad57*::*C.d.ARG4*).

CaLY244 (*MET3p-MMS22*/*mms22*::*C.d.HIS1 rtt101*::*C.m.LEU2*/*rtt101*::*C.d.ARG4*): *C. albicans* CaLY226 was transformed with *rtt101*::*C.m.LEU2* and *rtt101*::*C.d.ARG4* cassettes to generate strains CaLY240 (*MET3p-MMS22*/*mms22*::*C.d.HIS1 Rtt101*/*rtt101*::*C.m.LEU2*) and CaLY244 (*MET3p-MMS22*/*mms22*::*C.d.HIS1 rtt101*::*C.m.LEU2*/*rtt101*::*C.d.ARG4*).
