# Supplementary material for: The Role of Mms22p in DNA Damage Response in Candida albicans
Source: G3 (Bethesda). 2015 Oct 4;5(12):2567–78. doi: 10.1534/g3.115.021840 (PMC4683630; doi:10.1534/g3.115.021840)
Supplement: Supporting Information [file supp_g3.115.021840_TableS1.doc]

**Supplementary Table**

Table S1. The oligonucleotides used in strains construction.

| Strains | Primer | Primer sequence |
| --- | --- | --- |
| CaLY8 | P1 (oLY83) | CAAGACCATTTACAAGCAATCC |
|  | P3 (oLY84) | cacggcgcgcctagcagcggTTGAAAGTGGGAACAAGGTTAG |
|  | P4(oLY85) | gtcagcggccgcatccctgcCGATTCCAATTTGTCTTTGGC |
|  | P6(oLY86) | AACAAGAACCAGTCCCACC |
|  | UC(oLY87) | TGGTAAACTTATTCGTGCTGG |
|  | DC(oLY88) | ACAGCAGAAGACTTGAAAGAAC |
|  | IC1(oLY89) | ACGACGATTCAGATTCAAACC |
|  | IC2(oLY90) | GCTCTTCTTGAAGCTCTTTTTC |
|  |  |  |
| CaLY226 | oLY152 | CGCTTGGGCGACACTGTGGTGGCAAAGTAGTGCGACGTAGGTGCAAGTCTAAGACGAAGAAAAACTAGGGAAAGGCAAACGCGTCCAGCAATATTATTTTgaagcttcgtacgctgcaggtc |
|  | oLY153 | GATTATTATGAAAAATGCTAAAGTAGTGAATGAAACTGTGTTATATCTTTTAATATTATCATTAAGTTATGGTTCTATTTATATTTGAAAGTGGGAACAAcatgttttctggggagggtatttac |
|  | UC(oLY87) | TGGTAAACTTATTCGTGCTGG |
|  | DC(oLY88) | ACAGCAGAAGACTTGAAAGAAC |
|  | IC1(oLY89) | ACGACGATTCAGATTCAAACC |
|  | IC2(oLY90) | GCTCTTCTTGAAGCTCTTTTTC |
|  |  |  |
| CaLY219 | P1(oLY160) | TCGAGGATAGACCGTGAACC |
|  | P3(oLY161) | cacggcgcgcctagcagcggTTGAGAAGGCACAGCAACAG |
|  | P4(oLY162) | gtcagcggccgcatccctgcAAAAGAAACGCCCTGAACCT |
|  | P6(oLY163) | CTTTCACAGCTTTTGCCACA |
|  | UC(oLY461) | CAAGTTGGCTGGTGAAGTGA |
|  | DC(oLY462) | GGCGGAGACCATTGTGTAAT |
|  | IC1(oLY463) | GATCGAGAGTTGGCAGAAGG |
|  | IC2(oLY464) | GCTTGATGGAAAAACCTTGC |
|  |  |  |
| CaLY337 | P1(oLY534) | GAAACTTGGCTTGGGTCAAT |
|  | P3(oLY535) | cacggcgcgcctagcagcggAAAATCACCACGAACCCATC |
|  | P4(oLY536) | gtcagcggccgcatccctgcATGAGTGATTATGAATCAGG |
|  | P6(oLY537) | AGGCGGTGGTTGAATATCTG |
|  | UC(oLY461) | CAAGTTGGCTGGTGAAGTGA |
|  | DC(oLY462) | GGCGGAGACCATTGTGTAAT |
|  | IC1(oLY463) | GATCGAGAGTTGGCAGAAGG |
|  | IC2(oLY464) | GCTTGATGGAAAAACCTTGC |
|  |  |  |
| CaLY220 | P1(oLY465) | GGAGAGCAATTCTCCAGCAA |
| CaLY234 | P3(oLY466) | cacggcgcgcctagcagcggTGGAACGCGTTTATTATGGTC |
|  | P4(oLY467) | gtcagcggccgcatccctgcTGAGAGAGTACGGCGCATAA |
|  | P6(oLY468) | GGACAAGAGGTTTTCGGATG |
|  | UC(oLY469) | CGTCAATAGGTGGGCTGTTT |
|  | DC(oLY470) | TGACCAAACGCAAAAACGTA |
|  | IC1(oLY471) | TGTGGTGTCATGGCTTGTTT |
|  | IC2(oLY472) | CTGGGATACTTGGTGCGTCT |
|  |  |  |
| CaLY249 | P1(oLY543) | TCCTGTTATGCTTGCTTGTGA |
| CaLY246 | P3(oLY544) | cacggcgcgcctagcagcggCGTAATGGGAAGACGGAAAA |
|  | P4(oLY545) | gtcagcggccgcatccctgcATGTCATATGTGATGGACGA |
|  | P6(oLY546) | AACAAGCCATGACACCACAA |
|  | UC(oLY469) | CGTCAATAGGTGGGCTGTTT |
|  | DC(oLY470) | TGACCAAACGCAAAAACGTA |
|  | IC1(oLY471) | TGTGGTGTCATGGCTTGTTT |
|  | IC2(oLY472) | CTGGGATACTTGGTGCGTCT |
|  |  |  |
| CaLY222 | P1(oLY174) | AGATTGTTAGGAGGCGGTGA |
| CaLY228 | P3(oLY175) | cacggcgcgcctagcagcggTTTCACGACGTTTTTGTTCG |
|  | P4(oLY176) | gtcagcggccgcatccctgcAACCAAGGTGAAGAAGACGAAG |
|  | P6(oLY177) | ATTTTCATGGCCCCTCTTTT |
|  | UC(oLY178) | TGGCCATCAGGAAAGTTGA |
|  | DC(oLY179) | ACTGCTGGGAACCGATAATG |
|  | IC1(oLY180) | TGGCAATGGTGAAGATGAAG |
|  | IC2(oLY181) | TTTTACGACCACGACGAACA |
|  |  |  |
| CaLY316 | P1(oLY539) | ACAGTGATTGTCGTTTATTCAAGAG |
| CaLY251 | P3(oLY540) | cacggcgcgcctagcagcggATGCGAGCATCCCAATTCTA |
|  | P4(oLY541) | gtcagcggccgcatccctgcATGGATTTGTTAGATGGGAT |
|  | P6(oLY542) | CATCATCACCCTTGTCTTGG |
|  | UC(oLY178) | TGGCCATCAGGAAAGTTGA |
|  | DC(oLY179) | ACTGCTGGGAACCGATAATG |
|  | IC1(oLY180) | TGGCAATGGTGAAGATGAAG |
|  | IC2(oLY181) | TTTTACGACCACGACGAACA |
|  |  |  |
| CaLY223 | P1(oLY481) | ATGTTTGGGAGACGTGGTTG |
| CaLY235 | P3(oLY482) | cacggcgcgcctagcagcggGTCTCGTTCACACGAAAGCA |
| CaLY238 | P4(oLY483) | gtcagcggccgcatccctgcCAACCAACCAACGTGCTAGA |
| CaLY242 | P6(oLY484) | AAATTCTCTCGCAGTGCAGTC |
|  | UC(oLY485) | TCGTTTGAAAGACCACCACA |
|  | DC(oLY486) | TCGTTTTTCCCTCTCGATTG |
|  | IC1(oLY487) | TGGATTCAGACAAGGGGAAG |
|  | IC2(oLY488) | AATCAAGTTCTCCCGCCTCT |
|  |  |  |
| CaLY224 | P1(oLY489) | GGGTGTGAATCGAATGTATGAA |
| CaLY236 | P3(oLY490) | cacggcgcgcctagcagcggGATTGATGGGAAATGGGTTG |
| CaLY240 | P4(oLY491) | gtcagcggccgcatccctgcTCGACGTCCAGTAACTATGACAA |
| CaLY244 | P6(oLY492) | TTGGTTTTCTGGGGAGCTG |
|  | UC(oLY493) | GCCATTTTCCCCTTGTTTTT |
|  | DC(oLY494) | TTTGGTTTTCTGGGGAGCTG |
|  | IC1(oLY495) | ATCACTGTACCAACGGCAAA |
|  | IC2(oLY496) | CCCATTGTCATCTTCTGCTG |
|  |  |  |
|  | oLY232 | ccgctgctaggcgcgccgtgAGCTCGGATCCACTAGTAACG |
|  | oLY233 | gcagggatgcggccgctgacGCCAGTGTGATGGATATCTGC |
|  | oLY236 | CAAACACAACTGCACAATCTGGC |
|  | oLY237 | GATACGTTGGTGGTTCAGTTGAGG |
|  | oLY238 | TTACAAGTATGAAAGGAGGGG |
|  | oLY239 | CTTCAACCTTTCAAACGATGC |
|  | oLY300 | GCACACACTACTTAATATACACAGC |
|  | oLY301 | TCAAGTATACGTAATCTCCCC |
|  | oLY312 | ccgctgctaggcgcgccgtgGaagcttcgtacgctgcaggtc |
|  | oLY313 | gcagggatgcggccgctgacCatgttttctggggagggtatttac |
|  | oLY538 | TCATGCCATTCTTGTCTGAT |
|  | oLY366 | GCACGCCGTTACAGGAGTTA |
|  | oLY367 | GAAGTTGGTGACGCGATTGT |
